# Demographic processes constrain global growth in gender egalitarianism

**DOI:** 10.1093/pnasnexus/pgag133

**Published:** 2026-05-26

**Authors:** Plamen Akaliyski, Catherine E Bowen, Stefan Gehrig, Vegard Skirbekk

**Affiliations:** Department of Sociology and Social Policy, Lingnan University, 8 Castle Peak Road, Tuen Mun, Hong Kong SAR; Department of Demography, University of Vienna, 1010 Vienna, Austria; Independent Researcher, 12161 Berlin, Germany; Department of Psychology, University of Oslo, 0373 Oslo, Norway; Norwegian Institute of Public Health, 0456 Oslo, Norway

**Keywords:** gender equality, gender role beliefs, population shifts, differential fertility, demographic change

## Abstract

Women's and men's equal participation in work, education, and politics rests upon widespread public support for gender equality. While notable increases in support for gender equality over the past decades have been celebrated, existing evidence is limited by a predominant focus on Western societies. Utilizing data from the World Values Survey and European Values Study covering 78 countries that represent >86% of the global population, we examine how population-level support for gender equality in the public sphere changed between 1995 and 2022, both globally and across different cultural and socioeconomic contexts. Our results indicate that, although public support for gender equality increased in most countries, it stagnated in the global population as a whole. Moreover, there has been a pronounced and increasing divergence across countries, particularly between Western and non-Western societies. We attribute part of the stagnation in global public support for gender equality to demographically induced compositional effects. Namely, we find that (i) changes in the national composition of the global population have constrained increases in global public support for gender equality; (ii) fertility is higher in countries where average support for gender equality is lower; and (iii) fertility is higher and occurs earlier among women with less egalitarian views. Given that parents typically transmit their gender-related views to their children, higher and earlier fertility among persons with less egalitarian views has likely constrained global growth in gender egalitarianism. We call for a closer examination of how demographic processes influence global trends in gender egalitarianism.

Significance Statement While public support for gender equality has increased in many countries, it has stagnated globally. Higher population growth in countries exhibiting less public support for gender equality may have played a role in explaining global stagnation. Fertility—a key driver of population growth—is higher in countries with less public support for gender equality. It is also higher and occurs earlier among women with less egalitarian views. As parents tend to transmit their gender-related views to their children, the higher population growth and fertility of populations with less egalitarian views may undermine efforts to increase public support for gender equality. Our findings highlight the importance of demographic processes in shaping cultural change.

## Introduction

Women's and men's equal participation in education, paid work, and politics is seen as a key to improving individual and societal well-being (1–5; see also United Nations Sustainable Development Goal 5). As such, there has been great interest in monitoring societal support for gender equality in the public sphere. In high-income countries, public support for gender equality has increased dramatically in the past century (6–8). For instance, in 1936, just 18% of US Americans approved of a married woman working if she had a husband to support her. In 1985, it was 86% (9). The proportion of respondents who preferred a marriage with more traditional versus egalitarian gender roles in European countries dropped between 11 and 35 percentage points between 1991 and 2019 (10). There are, however, some indications that the trend toward greater gender egalitarianism in many high-income countries has stalled or even regressed since the turn of the century (8, 11–13). Moreover, the trends observed in high-income, predominantly Western countries may not necessarily reflect how beliefs about gender equality have changed in the world as a whole or in different world regions, which have cultural and socioeconomic contexts markedly distinct from those in the West (14).

In the current study, we examine how beliefs about gender equality in the public sphere (hereafter, gender role beliefs, GRB) have changed globally and in different world regions over the past three decades, specifically from 1995 to 2022. Our study is built on the premise that trends observed in Western—also known as WEIRD (Western, educated, industrialized, rich, and democratic) countries (15)—may not necessarily reflect those in the world as a whole. People living in Western countries constitute only a small proportion of the global population, and they differ in relevant ways from their peers in other world regions (e.g. with regard to education and religiosity, both of which are related to GRB (16)). Furthermore, societal GRB depend on numerous cultural and historical factors, including historical types of agriculture (e.g. dairy farming versus nomadic pastoralism and shifting cultivation (17)), language, geography, preindustrial societal characteristics, family structures, religion, and historical shocks (for a review, see (18)). Socioeconomic development is also thought to drive a shift from “traditional” to “modern” values emphasizing individual freedom and equal opportunity, including gender equality (19, 20). Hence, recent historical changes in GRB may vary across different cultural and socioeconomic contexts.

So far, there is surprisingly little empirical evidence on how GRB have changed globally. Analysis of two waves of the World Values Survey (WVS) covering 47% of the global population indicated that there was nearly no change in the proportion of global citizens with at least one bias concerning women in education, the labor market, politics, and women's physical integrity between 2010–2014 and 2017–2022 (86.9 to 84.6%) (21). Moreover, the proportion of people with at least one bias decreased in some countries and increased in others, indicating heterogeneity in the time trend across countries. In her descriptive analysis of data from five waves of the WVS from 1995 to 2022, Fike (22) found 52 countries with an overall upward and 23 countries with overall downward trend in GRB, with “not much of a discernable pattern in terms of *where* social norms [GRB] increased, decreased, or stayed fairly constant over time” (p. 18). Heterogeneous time trends in public support for gender equality in the public sphere were also observed in another study based on data from 1980 to 2020 (e.g. increasing in Norway and Australia; constant in South Korea and the Philippines; decreasing in Indonesia; increasing and then decreasing in Brazil; and decreasing and then increasing in Nigeria (23)). The observed heterogeneity in time trends across countries is consistent with evidence of a global divergence in societal values more generally (24, 25).

A second important yet understudied area of inquiry is how ongoing demographic dynamics may be impacting trends in GRB. Since 1995, average life expectancy has risen steadily and significantly across all regions, and fertility has fallen in most countries, but the timing and extent of these changes have varied widely (26). As a result, different countries and regions have experienced very different rates of population growth. While high-income countries have generally had slow or even negative population growth, low- and lower-middle–income countries have experienced rapid population growth (27). These different rates of population growth have changed the national composition of the global population. For example, the proportion of the global population living in North America and Europe shrank from 20.6% in 1995 to 16.9% in 2022, while the proportion living in Africa increased from 12.7 to 18.0% (26). Given that GRB tend to be more egalitarian in high- compared with low-income countries, unequal rates of population growth in high- and low-income countries may have impacted how GRB have changed in the world population as a whole.

“Differential fertility” may have also impacted trends in GRB. Certain ideologies may be reproduced more than others because (i) individuals’ fertility is associated with their ideologies and (ii) parents tend to transmit their ideologies to their children. In turn, the disproportionate reproduction of certain ideologies can influence trends in the cultural traits of the global and national populations over time. Recently, Vogl and Freese (28) provided evidence that higher fertility among more conservative individuals has contributed to the US population becoming less accepting of same-sex marriage and abortion. Similarly, differential fertility is making the global population more religious (29, 30).

People with more egalitarian GRB tend to have, or intend to have, fewer children (31–33). Fertility also tends to be lower in countries with stronger public support for gender egalitarianism, despite the tendency for reversal at very high public support for gender equality in the private sphere (i.e. the second phase of the so-called Gender Revolution (34, 35) and/or when egalitarian GRB are sufficiently widespread in society (6)).

Evidence has also accumulated that parents transmit their gender attitudes to their children (36–41) via a number of mechanisms, including socialization (37, 41, 42) and genetics (43, 44). Together, this evidence suggests that differential fertility may impact national and global trends in GRB. Of course, many other factors—economic development, educational expansion, institutional reforms, and international norm diffusion—may influence gender attitudes. However, we emphasize that differences in the fertility rates of more or less egalitarian countries may also contribute to unequal patterns of population growth.

To the best of our knowledge, the potential impact of differential fertility on global trends in GRB has not been explored. Moreover, we are unaware of research on differential “fertility timing” (i.e. the age at which a person has their first child), which may likewise impact patterns of cultural change. The offspring of younger mothers join the adult population (the focal population of many studies of cultural change) sooner than the offspring of older mothers. Thus, the gender ideologies of children born to younger mothers will impact the makeup of the adult population years earlier than the beliefs of children born to older mothers.

Our study addresses the dearth of evidence on global trends and regional variation in GRB. We extend previous research (21, 22) by employing an advanced modeling framework and using five waves of data from the WVS (45) and two waves from the European Values Study (EVS) (46). Our data cover the period from 1995 to 2022.^1^ We focus specifically on responses to three items concerning women's position in the public sphere: women's equal right to paid work, the equal importance of university education for boys and girls, and women's equal suitability as political leaders. By focusing on these three items that are relatively widely available in global data, we are able to capitalize on a sample of 78 countries that represent 86.1% of the 2022 world adult population (18+ years, i.e. the survey population). As in previous research using the same survey items (14, 20), we re-scale responses to each item to 0–100 and calculate the arithmetic mean of the three items as a measure of a person's GRB (Cronbach's *α* = 0.66 in the global sample). Higher scores indicate more egalitarian GRB. Analysis of the internal reliability and factor structure of the measure across cultural zones indicates that scores are comparable across contexts (see Supplementary Material S5.1). We use this measure to examine average trends in GRB in the world population (RQ1) and among populations of different cultural and socioeconomic contexts (RQ2). Longitudinal analyses of global surveys are often limited by the relatively sparse and unbalanced design. Data are missing for many years, the temporal spacing of surveys is heterogeneous, and the sample selection could be biased in different ways between surveys. We address these limitations by combining hierarchical generalized additive modeling (allowing flexible curve fitting and information borrowing) with poststratification (allowing sample representativeness adjustment for age and sex). We then explore whether differential population growth between nations partially explains the global trend (RQ3). Finally, to examine whether differential fertility plays a role, we explore how individual and national GRB are related to completed fertility and fertility timing (i.e. the typical age at which women become parents; RQ4).

## Results

### Global, regional, and country-specific changes in gender role beliefs, 1995–2022

**Figure 1:**
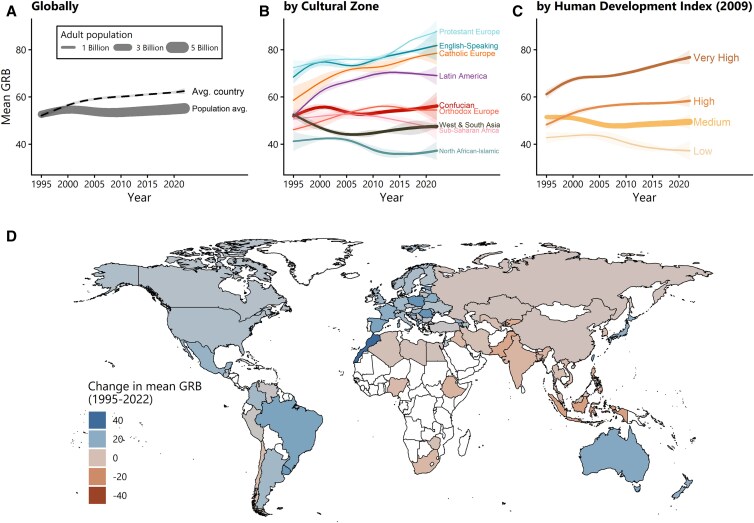
Gender role beliefs, 1995–2022. A higher score indicates more egalitarian GRB. A) Trends in global GRB in the average country (dashed line) and for the average global adult citizen (solid line). B) The GRB of the populations of different cultural zones. C) The GRB of more and less socioeconomically advanced populations as indicated by a country's HDI in 2009. All lines show posterior means. Shaded areas show pointwise 90% equal-tailed credible intervals. The thickness of the solid lines showing population averages indicates the represented adult population size (see legend in A). D) Point estimates (posterior means) of country-specific changes, with darker shades indicating larger changes.

Figure 1 displays trends in the average GRB score in the adult population of the world (A), of different cultural zones (B), of different socioeconomic contexts (C), and of individual countries (D). All panels present model-based estimates using data from the 78 countries with at least two survey waves that covered at least a quarter of the observation period.^2^ Figure 1A compares two metrics: a simple average of the global population with countries weighted equally (dashed line) and a population-weighted global average (solid line), which takes into account the proportion of the global adult population attributed to each country in the sample. The dashed line thus represents the trend in GRB in the average country, while the solid line represents the trend in GRB for the average global adult citizen. As indicated by the dashed line, the population in the average country steadily became more egalitarian from 1995 to 2022, increasing from 52.1 (50.9, 53.3; posterior mean with 90% equal-tailed credible interval) to 62.5 (61.3, 63.6) on a 100-point scale. The global population-weighted trend, however, tells a different story: according to this metric, global public support for gender equality in 2022 remained remarkably close to the 1995 level (from 52.7 [51.8, 53.5], reflecting the mean among 3.2 billion adults, to 55.2 [53.2, 57.1], reflecting the mean among 4.8 billion adults), with even a temporary drop in the early 2000s. The diverging trends suggest that, to some degree, the observed stagnation in global gender egalitarianism can be attributed to shifts in the relative “weight” of different national populations on the global average—or, in other words, differential rates of adult population growth.

Figure 1B reveals pronounced variation in both the starting levels and trajectories of GRB among the populations of different cultural zones (zones adapted from Welzel (20)). The populations of cultural zones covering countries conventionally classified as Western or WEIRD, i.e. Protestant and Catholic Europe and English-speaking offshoots in North America, Australia, and New Zealand, were, on average, more egalitarian in 1995 and generally became more egalitarian over time. Starting from a lower baseline, the population of Latin America initially followed the same trend toward greater egalitarianism as the WEIRD populations; however, since the early 2010s, it has become slightly less egalitarian. The population of historically Orthodox European countries followed a similar trend as the Latin American population, but starting from an even lower level. The population of the Confucian cultural zone, with China accounting for the largest population share, became only modestly more egalitarian during the observed period. On the other hand, the populations of the West and South Asia, Sub-Saharan Africa, and North African–Islamic cultural zones became less egalitarian. In sum, the populations of WEIRD cultural zones not only were more egalitarian in 1995 but also became even more so over time compared with the populations of other cultural zones. This has resulted in a significant divergence between GRB in WEIRD versus other cultural zones.

Figure 1C displays trends in GRB of more and less socioeconomically developed populations, as indicated by a country's Human Development Index (HDI: low, medium, high, and very high) in 2009, i.e. in the middle of the observation period. Figure 1C reveals that trends in GRB followed a clear socioeconomic gradient: in the very high HDI population, GRB were most egalitarian and became even more so over time. Gender egalitarianism was second highest in the high HDI population, making notable increases in the late 1990s, followed by near stagnation since the late 2000s. In contrast, the GRB of medium and particularly the low HDI populations fluctuated more often and became less egalitarian by 2022. As a consequence, the gap between the GRB of more and less socioeconomically advantaged populations has widened over time.

Finally, Fig. 1D maps point estimates of the country-specific trends in GRB (posterior mean difference in GRB between 1995 and 2022). As indicated by the diversity of color tones, there was substantial heterogeneity in both the direction and magnitude of change across national populations. The populations of many countries, including those in Western Europe, Latin America, and parts of East Asia, became notably more egalitarian. Others, particularly the national populations in parts of Sub-Saharan Africa, South Asia, and North Africa, experienced stagnation or even declines in egalitarianism.^3^ Estimated time trends for the populations of each country are presented in Supplementary Material S1.

### Differential population growth constrains increases in gender egalitarianism

**Figure 2:**
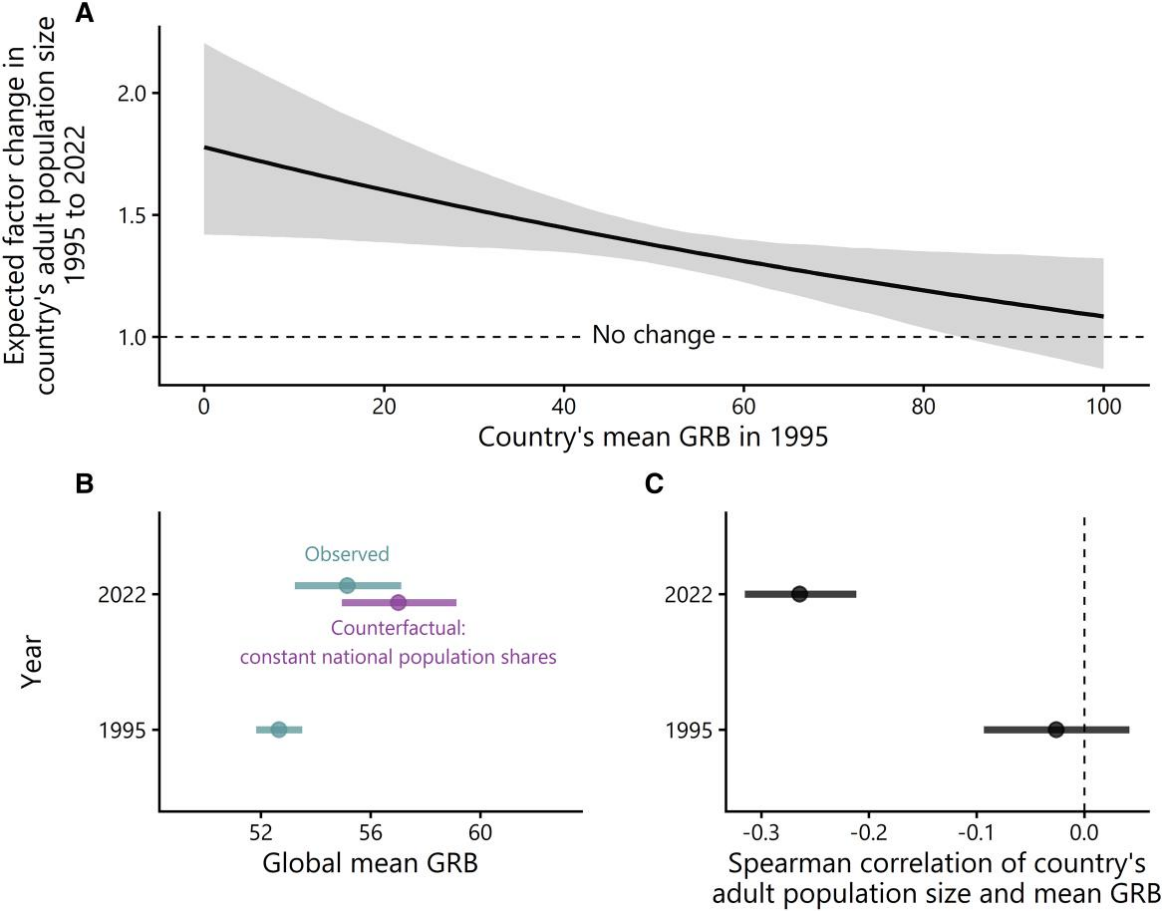
Demographic influences on gender role beliefs of the adult (18+ years) global population. A higher score indicates more egalitarian GRB. A) The estimated association between countries’ average GRB in 1995 and their subsequent rate of adult population growth in 1995–2022. B) Global trends in GRB as observed and the counterfactual, assuming a time-constant national composition of the global population. C) The correlations between countries’ adult population size and GRB in 1995 and 2022. Point estimates are posterior means. Intervals are 90% equal-tailed credible intervals.

Figure 2A presents the estimated relationship between the average GRB of a country's population in 1995^4^ and the country's subsequent adult population growth between 1995 and 2022. The figure reveals a clear negative association between GRB and population growth: countries with less egalitarian GRB in 1995 experienced greater adult population growth between 1995 and 2022 compared to countries with more egalitarian GRB. In other words, population growth has been disproportionately concentrated in countries with less egalitarian GRB. Figure 2B presents a simple counterfactual analysis that illustrates the impact of differential adult population growth on the global trend in GRB. The purpose of the counterfactual analysis is to illustrate what GRB trajectories would have looked like with and without the changes in the national composition of the global population that occurred between 1995 and 2022. Specifically, the figure compares the observed global trend (also shown in Fig. 1A) in GRB with what GRB would have been if, *ceteris paribus*, the national composition of the global population had remained constant at the levels observed in 1995 (counterfactual scenario). The comparison suggests that changes in the national composition of the global population constrained a global shift toward more gender egalitarianism. If the national composition of the global population had remained constant, global support for gender equality would have increased more strongly, from 52.7 (51.8, 53.5) to 57.0 (55.0, 59.1), as opposed to the observed 55.2 (53.2, 57.1).^5^ In other words, the increase in global support for gender equality would have been about 72% larger (posterior median of the relative difference in trends) than what was actually observed.

Correspondingly, Fig. 2C shows the association between countries’ population size and GRB at the beginning and end of the observation period. There was a weak, negative Spearman rank correlation between population size and GRB in 1995 (−0.03 [−0.09, 0.04]). That is, more populous countries tended to have slightly less egalitarian GRB. The negative association between population size and GRB was substantially stronger in 2022 (−0.26 [−0.32, −0.21]), indicating that the relative “weight” of countries with less egalitarian GRB on the global average has increased, in line with the pattern observed in Fig. 2A and B.

The results so far suggest that global GRB increased only minimally over almost three decades, even as the average country became more gender egalitarian. During the same period, a negative relationship between a country's mean GRB and its population size emerged: in contrast to 1995, more populous countries had lower mean GRB in 2022. In principle, this pattern could have arisen because more populous countries experienced smaller gains (or larger declines) in mean GRB. However, we find evidence that the observed trends are partly attributable to faster population growth in countries with lower GRB. Next, we examine how GRB is related to fertility as a potential mechanism linking GRB with population growth.

### Less egalitarian gender role beliefs are associated with higher and earlier fertility

To explore how differential fertility might constrain increases in global gender egalitarianism, we examined how GRB were related to women's fertility and fertility timing. In these analyses, rather than limiting our focus to regional or country-level aggregates, we investigated the covariation of GRB and women's fertility patterns not only between but also within countries. It was therefore possible to infer whether women with lower or higher GRB than their respective counterparts in the same cohorts exhibited, on average, different fertility patterns, thereby potentially contributing to population-level trends in GRB. Table 1 presents the results from cross-sectional linear mixed-effects models (LMMs) with women's self-reported number of children at age 40 to 49 as an outcome variable and both the country-average GRB and an individual woman's deviation from the country-average GRB included as fixed effects. The average number of children born to women aged 40 to 49 is an estimator of completed cohort fertility (47). Here, we used women's self-reported number of children at age 40 to 49 as a proxy of their completed fertility, that is, the number of births they have had toward the end of their reproductive window. The models’ specification allowed us to assess whether and how women's fertility was related to both the average GRB in their country and to their own GRB after accounting for the country-level effect.

**Table 1:** Results from LMMs for the number of children among women aged 40 to 49 years with country random intercepts and slopes.

		Model 1 (only GRB)		Model 2 (with demographics)		Model 3 (with demographics and religiosity)	
Term	Effect level	Estimate (SE)	*P*-value	Estimate (SE)	*P*-value	Estimate (SE)	*P*-value
Intercept		2.200 (0.058)	<0.001	2.849 (0.521)	<0.001	2.348 (0.514)	<0.001
GRB	Within	−0.102 (0.014)	<0.001	−0.053 (0.014)	<0.001	−0.044 (0.015)	0.004
GRB	Between	−0.622 (0.093)	<0.001	−0.362 (0.120)	0.003	−0.210 (0.118)	0.080
Age							
Age	Pooled			0.013 (0.004)	<0.001	0.012 (0.004)	0.002
Education							
Lower secondary	Within			−0.150 (0.042)	<0.001	−0.163 (0.043)	<0.001
Upper secondary	Within			−0.375 (0.038)	<0.001	−0.378 (0.039)	<0.001
Tertiary	Within			−0.529 (0.041)	<0.001	−0.537 (0.042)	<0.001
Lower secondary	Between			−1.588 (0.581)	0.008	−1.049 (0.550)	0.060
Upper secondary	Between			−1.544 (0.392)	<0.001	−1.121 (0.366)	0.003
Tertiary	Between			−1.161 (0.404)	0.005	−0.520 (0.401)	0.198
Income level							
Medium	Within			0.017 (0.027)	0.518	0.010 (0.027)	0.700
High	Within			0.129 (0.035)	<0.001	0.142 (0.036)	<0.001
Medium	Between			−0.261 (0.505)	0.607	−0.064 (0.473)	0.892
High	Between			0.059 (0.616)	0.924	0.114 (0.571)	0.842
Town size							
5,000–20,000	Within			−0.100 (0.035)	0.004	−0.106 (0.036)	0.003
20,000–100,000	Within			−0.191 (0.036)	<0.001	−0.192 (0.036)	<0.001
100,000–500,000	Within			−0.236 (0.038)	<0.001	−0.238 (0.039)	<0.001
>500,000	Within			−0.338 (0.039)	<0.001	−0.341 (0.040)	<0.001
5,000–20,000	Between			0.208 (0.538)	0.700	0.284 (0.493)	0.566
20,000–100,000	Between			0.347 (0.419)	0.409	0.149 (0.382)	0.698
100,000–500,000	Between			−0.474 (0.469)	0.315	−0.293 (0.427)	0.494
>500,000	Between			0.000 (0.319)	0.999	−0.014 (0.301)	0.962
Religiosity							
Religiosity	Within					0.099 (0.015)	<0.001
Religiosity	Between					0.365 (0.111)	0.001
Variance components							
Country intercept SD		0.574		0.504		0.459	
Country slope SD		0.050		0.047		0.048	
Residual SD		1.205		1.173		1.170	
Model information							
*N* obs.		14,283		12,644		12,016	
*N* countries		101		96		95	
Cond. *R*^2^		0.250		0.255		0.252	

GRB and religiosity are scaled to a mean of zero and an SD of 1. Reference categories are primary or less (education), low (income), and <5,000 (town size). Within effects refer to predictors that were mean-centered per country. Between effects refer to the country’s means as predictors. Age was modeled as a pooled within-between effect since the data were already restricted to the same age range across all countries. Estimates are shown with standard error (SE) in parentheses.

The analysis drew on 101 countries, using only the most recent survey per country, conducted no earlier than 2010. In additional models, we entered further predictors related to demographics (age, education, income, and town size) and religiosity, again both on the individual and country level. The models included random intercepts and random slopes for the effect of GRB to account for unobserved between-country variation. The results reveal a robust, negative association between women's GRB and their average number of children at age 40 to 49: women with more egalitarian GRB have fewer children (−0.10 [−0.13, −0.08; 90% CI] for a 1 standard deviation (SD) increase in GRB, equal to ∼28 points). Moreover, women's number of children at age 40 to 49 is negatively associated with their country's average GRB: in countries with more egalitarian GRB, women tend to have fewer children at age 40 to 49 (−0.62 [−0.78, −0.47] for a 1 SD increase in country-average GRB). Adjusting for demographics (age, education, income, and town size) and religiosity reduces, but does not completely account for, the within- and between-country relationships between GRB and number of children at age 40 to 49. We not only replicate the strong between-country association of societal religiosity and fertility rate demonstrated previously (30), but also extract a within-country religiosity effect, as well as within- and between-country effects of gender attitudes beyond what is captured by religiosity. Predictions from the estimated mixed-effects model are visualized in Supplementary Material S2.

**Figure 3:**
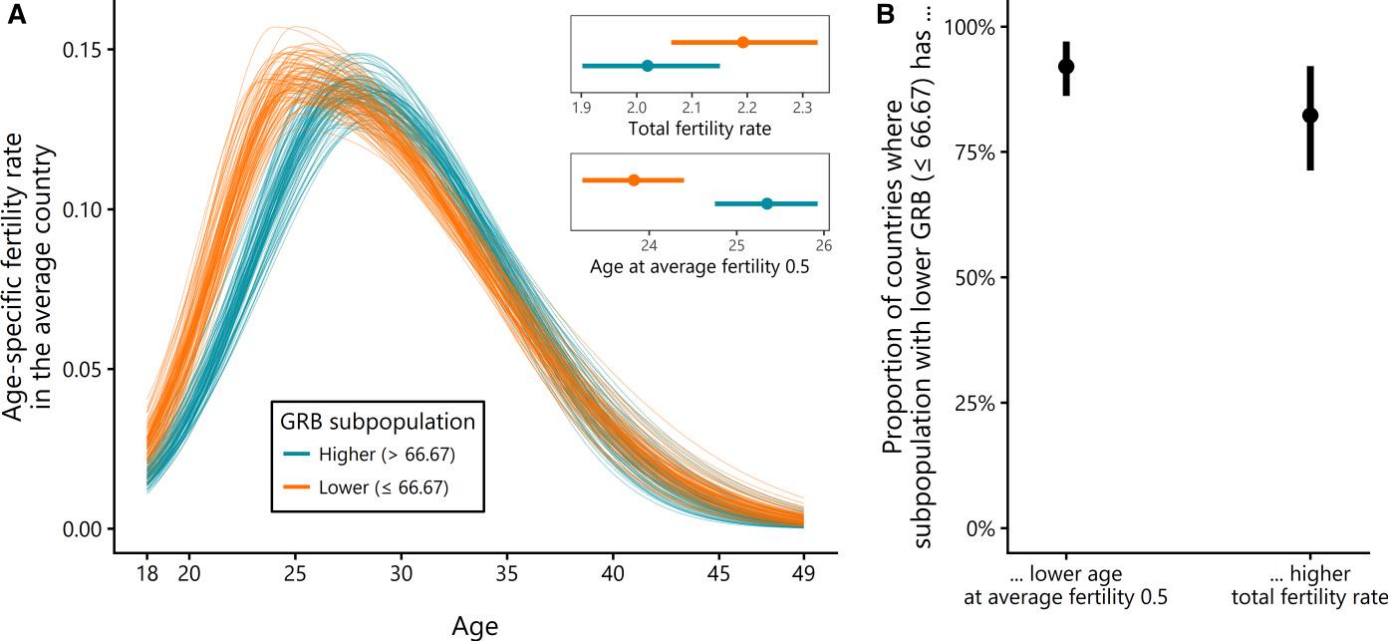
Age-specific fertility and gender role beliefs. A) Age-specific fertility rates for women with equal or lower (less egalitarian) versus higher (more egalitarian) than the global median GRB of 66.67 in the average country (all country-varying model parameters set to their mean value). The multiple lines represent 100 random draws from the posterior distribution. The insets show metrics derived from the curves: total fertility rate (i.e. area under the curve) in the upper inset, and age at which average fertility of 0.5 per woman is reached (i.e. age at which area under the curve equals 0.5) in the lower inset. B) By using the same metrics, the image shows the estimated proportion of countries in which women with less egalitarian GRB have earlier and higher fertility, based on country- and age-specific fertility curves (Supplementary Material S3). Points with intervals indicate posterior mean and 90% equal-tailed credible intervals, respectively.

Figure 3 presents the results of a hierarchical period age-specific fertility rate model, drawing on the same cross-sectional dataset of surveys and variables (i.e. again using the reported number of children as a proxy for the number of children ever born), but including women aged 18 to 49. This allows the estimation of age-specific fertility rates of women with less and more egalitarian GRB. These age-specific rates usually differ considerably between countries, and we hence allowed country-varying parameters as part of a hierarchical model. The resulting age-specific fertility curves for each country (see Supplementary Material S3) allow examination of differences in average fertility timing and total fertility rate between women with less and more egalitarian GRB in the same society.

Figure 3A displays estimated fertility curves. The curves depict the age-specific fertility rates of two subpopulations, women with GRB equal or lower versus higher than the global sample median (global sample median: 66.67 in the all-female sample from 101 countries used for modeling) in the average country (multiple lines are multiple posterior draws, illustrating statistical uncertainty). Here, the average country refers to the hypothetical country where all country-varying model parameters are set to their mean value. Figure 3B summarizes the extent to which the patterns observed in the average curve in Fig. 3A hold across individual countries. The results show that women with less egalitarian GRB begin childbearing earlier. Specifically, the average age at which one child per two women (i.e. average fertility 0.5) is born is 1.52 years lower (1.15, 1.90; posterior mean with 90% equal-tailed credible interval) among women with less egalitarian GRB. Also, women with less egalitarian GRB have higher total fertility compared to their peers with more egalitarian GRB (difference: 0.17 children [0.09, 0.26]). In terms of effect size, the fertility differential we observe is roughly comparable to the gap between Northern Europe—often seen as highly conducive to childbearing due to supportive family policies—and Southern Europe (1.5 versus 1.3 children per woman, respectively (26)) and exceeds the estimated effect of an additional year of schooling on the total fertility rate in Sub-Saharan Africa (48). The ranking of the two subpopulations in terms of the two assessed fertility characteristics was consistent across the vast majority of countries (92.0% [86.1, 97.0%] and 82.3% [71.3, 92.1%], respectively, see Fig. 3B). Together, these findings suggest that earlier and higher fertility among women with less egalitarian GRB may systematically constrain increases in global gender egalitarianism over time.

## Discussion

Our study addresses the dearth of evidence regarding global trends in GRB. Using data from 78 countries covering over 86% of the global population, we find that GRB became more egalitarian in most countries between 1995 and 2022. Nevertheless, the shift toward greater egalitarianism in most countries has not translated into a corresponding increase in global gender egalitarianism. Global gender egalitarianism has largely stagnated. Moreover, there is a growing divergence, particularly between WEIRD- and non-WEIRD populations, as well as between more and less socioeconomically developed populations. Our study extends limited earlier observations of stagnation in GRB (21) and is consistent with evidence of a global divergence in societal values (25).

Our analyses suggest that differential population growth and differential fertility may at least partially explain why global support for gender equality has stalled, despite a shift toward greater egalitarianism within the majority of countries. Over the past three decades, the populations of countries with less egalitarian GRB in 1995 grew faster than those in countries with more egalitarian GRB. We find evidence that changes in the national composition of the global population had a marked impact on how global GRB have changed in the past three decades. Our results also confirm the relationship between GRB at both the individual and societal levels and fertility (28, 30–32, 34, 35) and present a novel finding about a corresponding association with fertility timing, adding to existing research by considering fertility timing alongside fertility levels. Taken together, our results suggest that differential population growth and differential fertility have constrained global increases in gender egalitarianism. The higher and earlier fertility observed in countries with less egalitarian GRB may have promoted higher population growth.

Our study contributes to a growing body of work that adopts a differential demographic perspective to explain the persistence of traditional and conservative values (28, 49, 50). So far, this line of research has mainly focused on WEIRD societies, particularly the United States and Europe, where fertility differences between people with more or less conservative values and attitudes have been linked to cultural stasis or backlash. Our study suggests that differential population growth and differential fertility impact global trends in GRB. Our study contributes to the growing literature on global patterns of cultural change and divergence (24, 25, 51), showing that demographic processes may play a central role in shaping the uneven pace of cultural change worldwide.

We propose that differential demographic mechanics should be considered alongside other dominant theories of cultural change, such as modernization theory (19), supranational identity and cultural diffusion (52), and institutional accounts that emphasize the role of socioeconomic development, education, media, and policy environments (53). Existing approaches focus primarily on shifts in individual attitudes due to a sense of existential security, exposure to new ideas, or structural changes, but they tend to overlook more mechanistic demographic processes. Previous research has proposed cohort replacement—that is, the replacement of older (presumably more conservative) by younger (presumably more egalitarian) cohorts—as the primary mechanism driving long-term value change in general (54), and GRB in particular (12, 55). Our results suggest that fertility patterns not only influence the size of populations or the speed of cohort replacement but also which belief systems are more likely to be reproduced and persist over time. When individuals or groups with particular ideologies, such as those with less egalitarian GRB, consistently have more children, have them earlier in life, and transmit those ideologies to the next generation, demographic reproduction turns into a powerful cultural force (28, 30, 41). As such, “differential” demography offers a compelling and underutilized lens for understanding patterns of cultural change.

Importantly, differential demography can coincide with other mechanisms of attitude change, and the net effect of compositional shifts on global GRB depends on broader secular trends and structural transformations. In particular, educational expansion may attenuate compositional effects through its presumed impact on both fertility decline (56) and more egalitarian GRB (57). Indeed, our results indicate that controlling for education and other sociodemographic variables approximately halves the magnitude of the relationship between GRB and fertility at the individual level (Table 1). Given its relatively low GRB (according to our estimates based on four countries) and comparatively high fertility rates, how global GRB changes in the future may be disproportionately driven by trends in Sub-Saharan Africa.

### Limitations and future research

Our study covers a large share of the global population. Our results, nevertheless, are limited by the underrepresentation of regions such as Africa and the Middle East in our data—regions where public support for gender equality tends to be low and where population growth has been high (58–60). As a result, we speculate that we have likely underestimated the true extent of global stagnation. We also focused specifically on public support for gender equality in the public sphere (work, education, and politics). We were unable to investigate public support for gender equality in the private sphere of home and family due to a lack of adequate data. Research has increasingly recognized that GRB concerning the public sphere are largely independent from GRB concerning the private sphere (8, 35, 61–63). Future research should expand data coverage in Africa and the Middle East and include items capturing GRB in the private sphere.

We used data on women's self-reported number of children as a proxy for their fertility. Because respondents presumably report children currently living rather than births, this measure may underestimate fertility in contexts with elevated child mortality. If child mortality is higher in such settings and among social groups characterized by less egalitarian GRB, our results would understate the association between GRB and fertility. We also used a self-reported measure of GRB. Our results may thus be affected by the social desirability of gender egalitarianism, which may potentially vary across cultural contexts and time. If the normative pressure to endorse egalitarian views has increased over time, our results may underestimate the extent of global stagnation in GRB. Given that the normative pressure to endorse egalitarian views is apt to differ across societies, our results may exaggerate the heterogeneity in privately held gender attitudes between countries and cultural regions. We note that normative pressures constitute an important component of culture and may shape real-world behavior, potentially making them as consequential as privately held beliefs (64). Future research using alternative measurement strategies could further disentangle normative compliance from privately held gender attitudes.

Our results primarily describe trends and patterns but we cannot prove any relationships were causal. Given that education is positively related to GRB (16), higher fertility among people with less education compared to those with more education (65), as also reflected in our results (Table 1), might have contributed to stagnation in public support for gender egalitarianism. Other demographic dynamics, particularly differential mortality and migration rates, also warrant consideration. Differential mortality related to public and individual support for gender equality might, for instance, attenuate or offset any effect of differential fertility, especially in low-income settings where both traditional values and health burdens are more prevalent (66). Migrant flows tend to move from less to more gender egalitarian countries (67). While first-generation migrants may come from relatively liberal segments of their origin populations, their GRB are likely less egalitarian than those in host societies, potentially contributing to GRB stagnation even in highly developed countries (68). Future research should consider how mortality, migration, and other demographic dynamics shape long-term trends in GRB, as well as compare the relative impact of demographic mechanisms with other mechanisms (e.g. socioeconomic development, structural changes, and diffusion).

Another limitation is that our analysis did not directly demonstrate the intergenerational transmission of GRB from parents to children. By now, many studies have provided robust evidence that the gender attitudes of offspring tend to resemble those of their parents (36–44). However, we cannot observe this process directly in our data. We acknowledge that parents are hardly the only source of a person's gender ideologies, which also change throughout life and in response to different life experiences (69–72). Methodologically, the statistical models we apply to the global survey datasets rely on multiple modeling assumptions. Most importantly, we estimated smooth time trends in average GRB from year 1995 to year 2022 for every country and age-sex group in one hierarchical generalized additive model. Given that only between 2 and 5 survey years are available per country for this period (median: 3), most years were implicitly imputed by the model-fitting procedure, which borrowed information across time and countries in the same cultural zone. However, we note that the resulting estimated country-specific trends (see Supplementary Material S1) are much more plausible than results from previously used ad hoc methods in global survey research like linear interpolation or last-observed-value-carried-forward imputation. Further, the models automatically accounted for the increased uncertainty in a country's mean GRB for the years with missing survey data. We also note that the results were not sensitive to alternative inclusion criteria (see Supplementary Material S6).

Lastly, poststratifying on age and sex with external census data accounted for any potential survey sampling bias with respect to these two characteristics, but other attributes (e.g. urban/rural disparities) could not be addressed. For such attributes, it is possible that the sampled population shifted over the decades (e.g. becoming less urban due to increased accessibility of rural areas). Still, the survey networks whose data we used put great effort into ensuring nationally representative samples and are well-trusted in this regard. Future research should attempt to include further variables hypothesized to covary with GRB in their poststratification scheme.

## Materials and methods

Here, we provide a summary of the statistical methods. More methodological background and technical details are provided in Supplementary Material S5. All analyses were conducted in R v4.5.2 (73) and can be publicly accessed at https://github.com/stefgehrig/globalgender.

For research questions RQ1 to RQ3, the workhorse of our analysis was a Gaussian hierarchical generalized additive model (HGAM; 74), further described in Supplementary Material S5.2. The HGAM has become a tool of choice for modeling large-scale, longitudinal survey datasets with multiple missing country-year combinations, and can be used together with poststratification (75). Our model estimated a flexible, smooth effect of time on mean GRB separately for each cultural zone, as well as separately for each country, shrunk toward their cultural zone's average trend. The group-wise smoothers for cultural zones and countries were modeled with hierarchical factor-interaction splines implemented in the *mgcv* package (74, 76). Besides shrinkage toward the mean trajectory of countries in the cultural zone, this induces a similar level of smoothness for all countries’ time effects: One common smoothing parameter penalizes the “wiggliness” of the country-specific curves. Hence, missing survey years for countries led to the borrowing of information across neighboring time and neighboring cultural space, associated with an increase in uncertainty reflected in estimated variances (see Supplementary Material S1). We fitted the model to 373,855 observations from 78 countries, collected in 270 national surveys within the observation period from 1995 to 2022 (the national surveys with sample sizes are listed in Supplementary Material S5.2). In addition to country random intercepts, the model also included fixed effects for six demographic groups resulting from interacting age and sex ([18–34 years; 35–59 years; 60+ years] × [male, female]), as well as random, country-specific deviations from these age-sex group effects (random slopes). Estimates of the country-specific demographic-group effects are visualized in Supplementary Material S4.

We were ultimately interested in the time trend in the population mean GRB (e.g. globally), which is sensitive to the national and demographic composition of the population, and changes therein. Therefore, the representativeness of the survey samples continuously over time was important. To achieve this, after model fitting, we poststratified on the six age-sex groups defined above relying on UN's World Population Prospects (26) data on current and historical annual national population sizes by age and sex. The present use case, where subgroup effects were estimated in a hierarchical model and then combined according to their population shares, is an example of the “multilevel regression and poststratification” approach (77, 78). This approach has previously been applied to survey data in the context of smooth group-specific time trend estimation (75, 79). We computed a poststratified national mean GRB for each year and each of the 2,000 posterior draws from the model (obtained by sampling from the multivariate normal approximation of the model's joint posterior). In this way, point estimates of population mean GRB time trends and their associated uncertainty were obtained for countries (Supplementary Material S1). Further aggregation allowed the same for cultural zones, for HDI groups, or for the whole world, each time weighted according to national adult population shares, delivering results for RQ1 and RQ2.

For addressing RQ3, we again relied on the posterior draws of country and global mean GRB, obtained from the HGAM with poststratification, but only for the years 1995 and 2022. The counterfactual (Fig. 2B) and Spearman correlation analyses (Fig. 2C) were directly computed from the HGAM results and population size data. We estimated the relationship between a country's mean GRB in 1995 and its population growth from 1995 to 2022 (Fig. 2A) using a linear model for the log-transformed population data. Uncertainty about a country's mean GRB in 1995, reflected in the spread of poststratified posterior samples from the HGAM, was included in the form of measurement error in the model specification (log adult population 2022 as the outcome variable, log adult population 1995 as the offset variable, mean population GRB as predictor with measurement error). We fit the model with the *brms* package's function for Bayesian measurement error models and with weakly informative priors (80). After fitting, the specification allowed a simple transformation of the original outcome variable into population size factor change.

For RQ4, we used two modeling approaches with cross-sectional individual-level data, including only the most recent national survey that could be obtained for each country (but not older than 2010 to ensure that samples come from approximately the same time period). First, we fit an LMM, further described in Supplementary Material S5.3. In the LMM, the number of children reported by a woman was the outcome variable. The sample was restricted to women and to the age range of 40 to 49 years, as the goal was to approximate the number of children toward the end of a woman's reproductive years. Although GRB are measured after most childbearing has occurred, the temporal lag between the measure is unproblematic, as parental attitudes continue to be transmitted to their offspring throughout childhood and adolescence. The sample mean of GRB in a woman's country was included as a predictor along with a woman's deviation from it. Random country intercepts and random country slopes for GRB were included to account for unobserved variation between countries (i) in fertility and (ii) in the association between GRB and fertility. This approach, combining group-mean centering and random effects, has been called the “hybrid model,” as it provides advantages of both fixed- and random-effects estimation (81). Most importantly, it allowed decomposing within- and between-country effects of GRB. We extended the model with further predictors commonly assumed to be associated with fertility and/or gender equality values or both, again splitting them into within- and between-country components via group-mean centering. All continuous variables were standardized to have a mean of zero and a SD of one on the individual respondent level before mean centering them by country. Respondents with missing data for a variable were removed. One of the additional predictors was religiosity, measured as the arithmetic mean of four survey items related to religious values and behavior (see Supplementary Material S5.3), after rescaling each to a 0–100 scale.

Second, we fit a Bayesian hierarchical period age-specific fertility rate (ASFR) model, further described in Supplementary Material S5.4. Whereas the LMM yielded estimates of within- and between-country effects of GRB on the average number of children, the ASFR model allowed to compare full estimated fertility curves between a low-GRB and a high-GRB subpopulation, comparing age-specific fertility at various ages. The subpopulations were defined by a global sample median split. All countries contributed observations for both subpopulations (equal or lower and higher than median), and country-level subpopulation sample sizes are reported in Table S2. Note that the hierarchical structure of the ASFR model stabilized estimation in countries with smaller samples through partial pooling across countries and subpopulations, with remaining uncertainty fully reflected in the posterior distributions (see Supplementary Material S5.4). The number of children a woman reported having was used as a proxy for her fertility at a given age. The sample was restricted to women in the age range 18 to 49 years in order to adequately reflect period fertility. Specifically, we adapted the nonlinear, four-parameter ASFR model from Peristera and Kostaki (82). Its parameters can be interpreted in terms of (i) a “scaling factor” for the height of the fertility curve, (ii) a location of its peak (i.e. age with highest fertility), and a differential steepness of the curve (iii) before, and (iv) after the peak (Fig. 3A). It was assumed that the number of children followed a Poisson distribution, with the mean parameter being conditional on the age of the woman; specifically, being the sum of all ASFR values at ages prior to or at the woman's current age (i.e. her lifetime accumulated ASFR). Two fertility curves were fit, one for each subpopulation in terms of GRB, resulting in eight parameters to be estimated per country. The estimation was embedded in one global Bayesian hierarchical model, which induced shrinkage toward a common mean value for each country-specific model parameter. This was a desirable property, given that some GRB subpopulations in some countries had small samples (see Supplementary Material S5.4). The model was fit in Stan (83) with the *cmdstanr* interface (84), using weakly informative prior informed by the previous demographic literature (82). From the samples of the joint posterior distribution of the ASFR model's parameters, we could derive quantities like a country's and subpopulation's estimated fertility curves (see Supplementary Material S3), the posterior distribution of their total fertility (area under the curve), or the posterior distribution of their age at which average fertility equaled 0.5 (age at which area under the curve equals 0.5). Analogously, we derived the posterior distribution of the proportion of countries in which the low-GRB subpopulation had higher fertility, and a younger age at which average fertility reached 0.5.

## Supplementary Material

pgag133_Supplementary_Data

## Data Availability

The data used in this study are drawn from the publicly available World Values Survey and European Values Study datasets, accessible at https://www.worldvaluessurvey.org/ and https://www.europeanvaluesstudy.eu. The harmonized dataset and code used for analysis in this study are publicly available on GitHub at https://github.com/stefgehrig/globalgender.

## Notes

1 Earlier WVS and EVS waves lacked the data necessary for constructing our indicator of GRB.

2 A sensitivity analysis with varied sample inclusion criteria (minimum number of survey waves and temporal coverage) is provided in Supplementary Material S6.

3 One exception to the pattern is Morocco. However, the data for Morocco were scarce, and the estimate of the change remains highly uncertain, which is not visible in the figure showing only point estimates of change.

4 The mean GRB of a country’s population in 1995 was not directly observed and hence was modeled as a predictor with measurement error.

5 The wider credible intervals for year 2022 than year 1995 in Fig. 2B can be explained by fewer countries being sampled in that year and the larger global variance of GRB in that year (Fig. 1B).
