# Supplementary material for: Demographic processes constrain global growth in gender egalitarianism
Source: PNAS Nexus. 2026 May 26;5(5):pgag133. doi: 10.1093/pnasnexus/pgag133 (PMC13203941; doi:10.1093/pnasnexus/pgag133)
Supplement: pgag133_Supplementary_Data [file pgag133_supplementary_data.pdf]

Supplementary Material for
"Demographic processes constrain global growth in gender egalitarianism"

Plamen Akaliyski, Catherine E. Bowen, Stefan Gehrig, Vegard Skirbekk

Publication: https://doi.org/10.1093/pnasnexus/pgag133
GitHub (dataset and code): https://github.com/stefgehrig/globalgender

Contents

S1 Country-specific trends in mean GRB **1**

S2 Association of GRB and number of children **9**

S3 Country-specific cumulative age-specific fertility rates **10**

S4 Country-specific demographic effects on GRB **18**

S5 Additional methodological details **19**
S5.1 Measurement of GRB . . . 19
S5.2 The HGAM and post-stratification . . . 23
S5.3 The LMM . . . 28
S5.4 The Bayesian hierarchical period ASFR model . . . 30

S6 Sensitivity analysis results **36**

References **37**

S1 Country-specific trends in mean GRB

Figure S1 shows the estimated time trends in mean gender role beliefs (GRB) for all 78 countries included in the longitudinal analysis, with one panel for each cultural zone. These estimates were obtained from the hierarchical generalized additive model (HGAM) with post-stratification on age and sex groups (see Supplementary Materials S5.2).

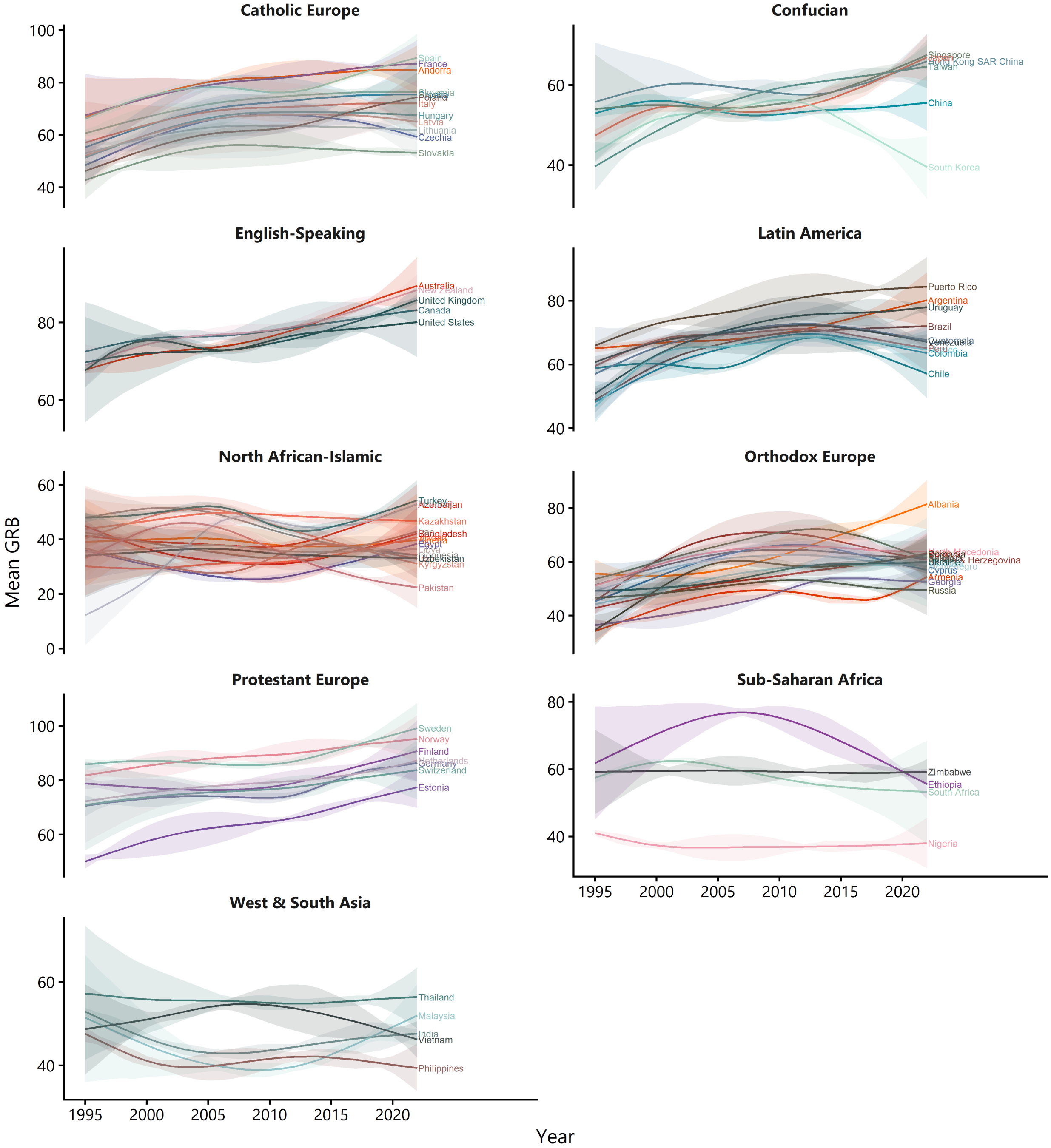

Figure S1: Estimated mean GRB over time of the populations of different countries from the HGAM with post-stratification (point estimate is the posterior mean) with pointwise 90% equal-tailed credible intervals.

In Figures S2 to S10, we plot these estimated longitudinal trends of mean national population GRB one-by-one and together with the raw (unweighted) national survey sample means. It can be seen that the HGAM with post-stratification delivers estimates mostly indistinguishable from the sample means in years where there is a survey, but provides flexible inter- and extrapolation with shrinkage towards the cultural zone average trend when there is no survey – along with a substantial increase in uncertainty.

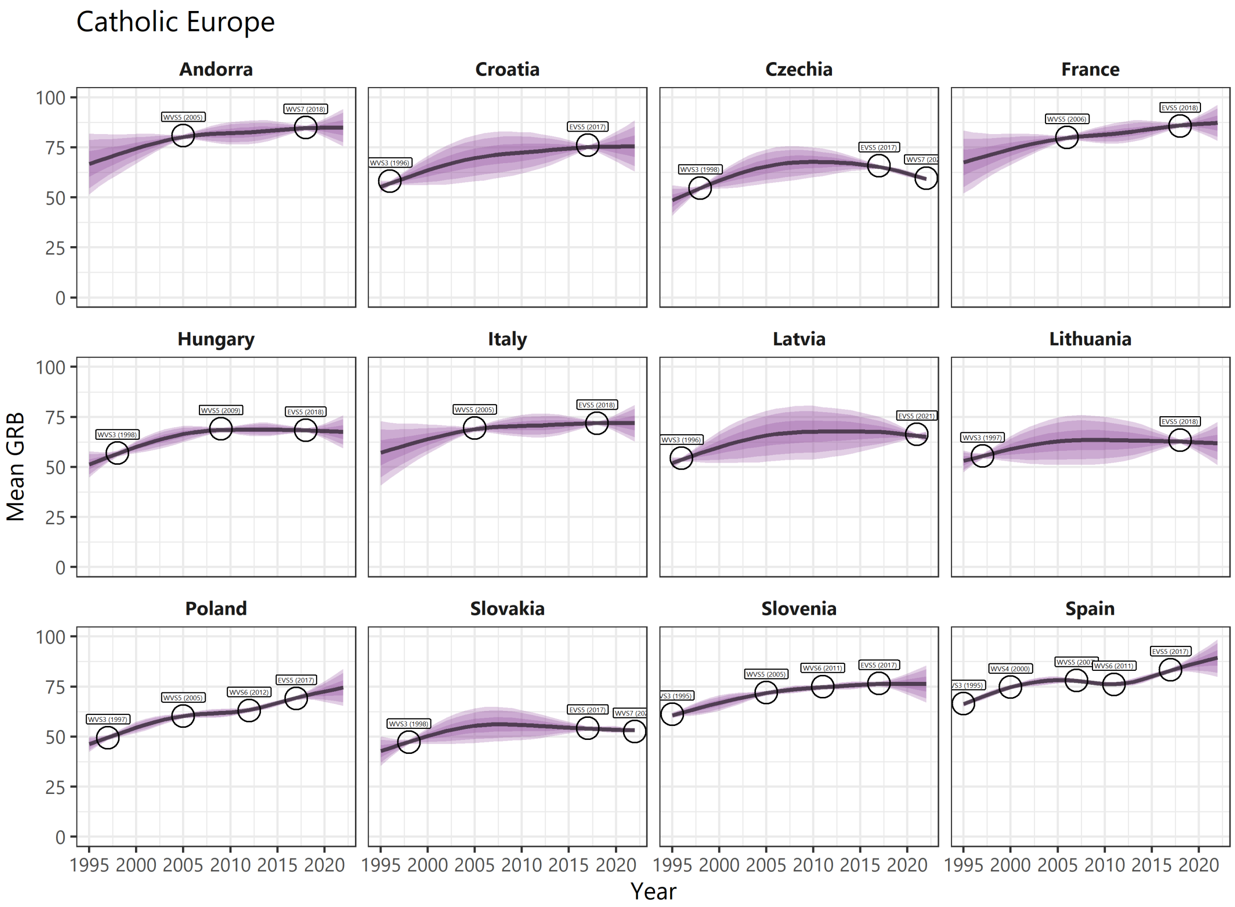

Figure S2: Estimated mean GRB over time of the populations of countries in the cultural zone *Catholic Europe*. Lines are estimates (posterior means) from the HGAM with post-stratification. Pointwise 50%, 80%, and 90% equal-tailed credible intervals are shown in purple. Points are sample means in the survey wave and year noted above each point.

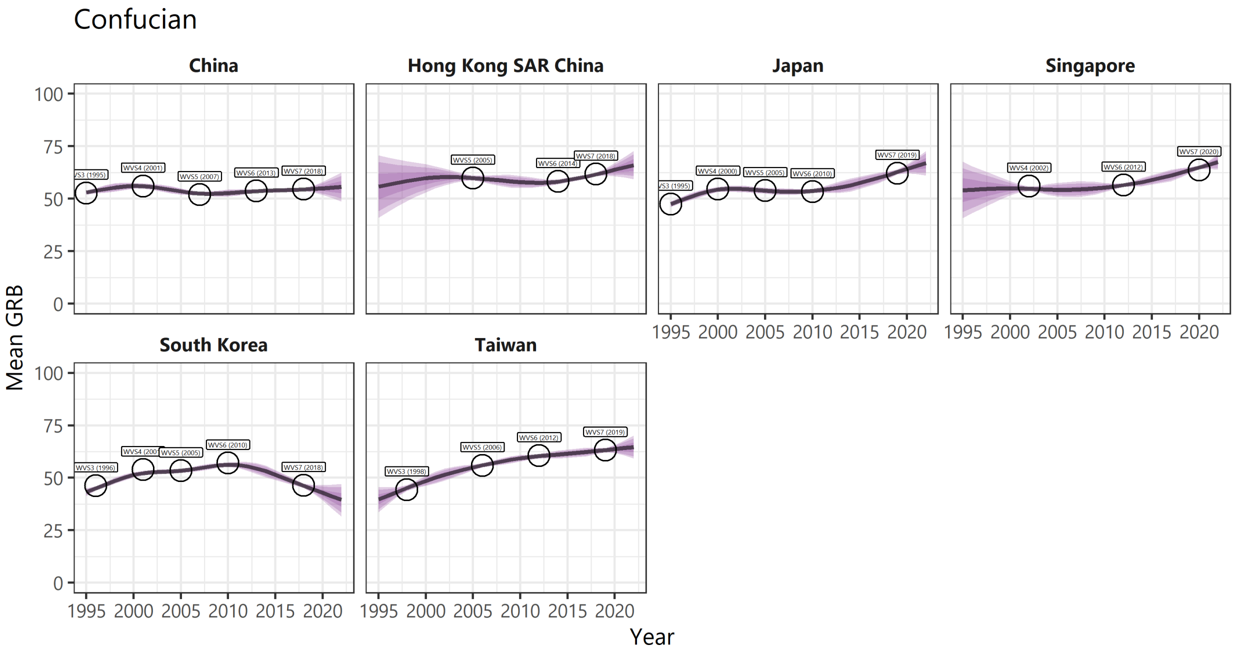

Figure S3: Estimated mean GRB over time of the populations of countries in the cultural zone *Confucian*. Lines are estimates (posterior means) from the HGAM with post-stratification. Pointwise 50%, 80%, and 90% equal-tailed credible intervals are shown in purple. Points are sample means in the survey wave and year noted above each point.

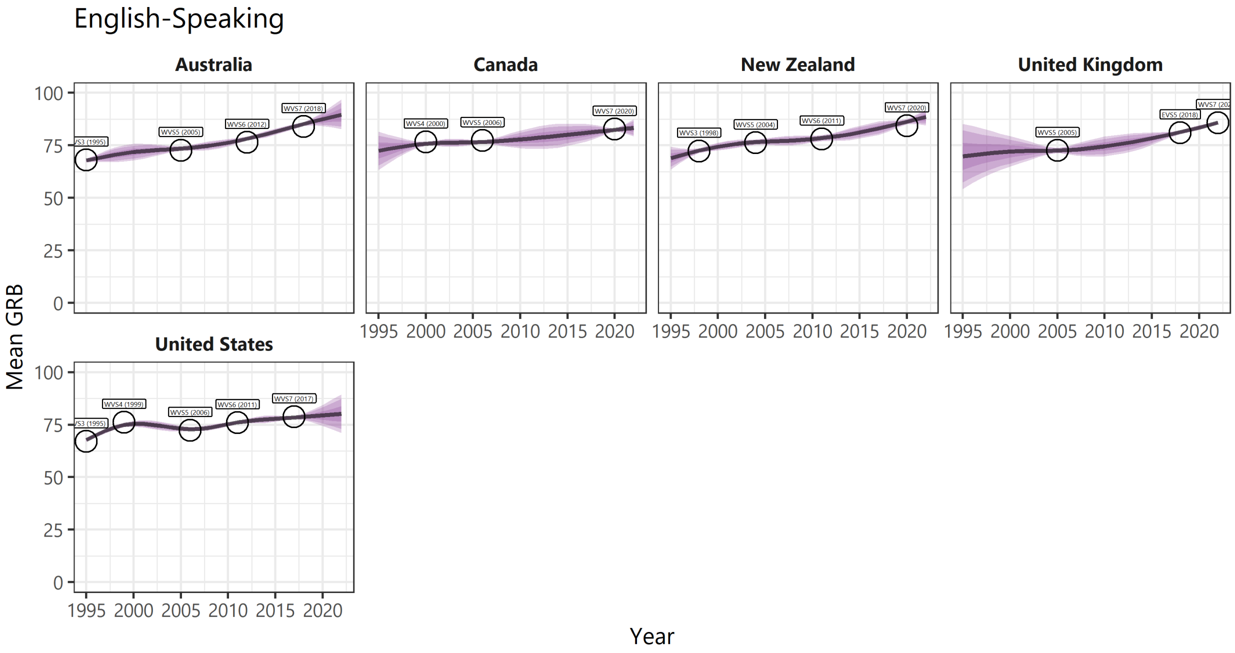

Figure S4: Estimated mean GRB over time of the populations of countries in the cultural zone *English-Speaking*. Lines are estimates (posterior means) from the HGAM with post-stratification. Pointwise 50%, 80%, and 90% equal-tailed credible intervals are shown in purple. Points are sample means in the survey wave and year noted above each point.

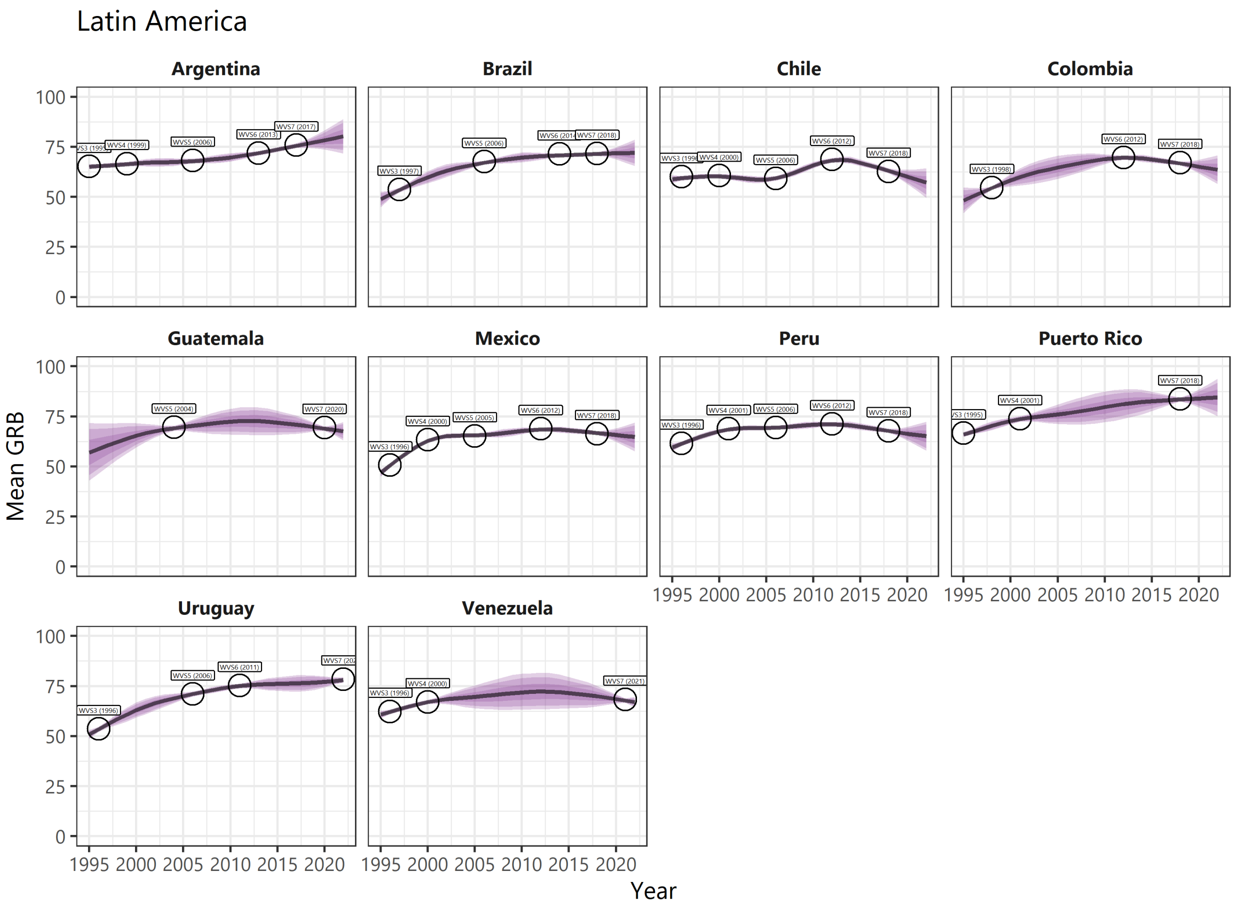

Figure S5: Estimated mean GRB over time of the populations of countries in the cultural zone *Latin America*. Lines are estimates (posterior means) from the HGAM with post-stratification. Pointwise 50%, 80%, and 90% equal-tailed credible intervals are shown in purple. Points are sample means in the survey wave and year noted above each point.

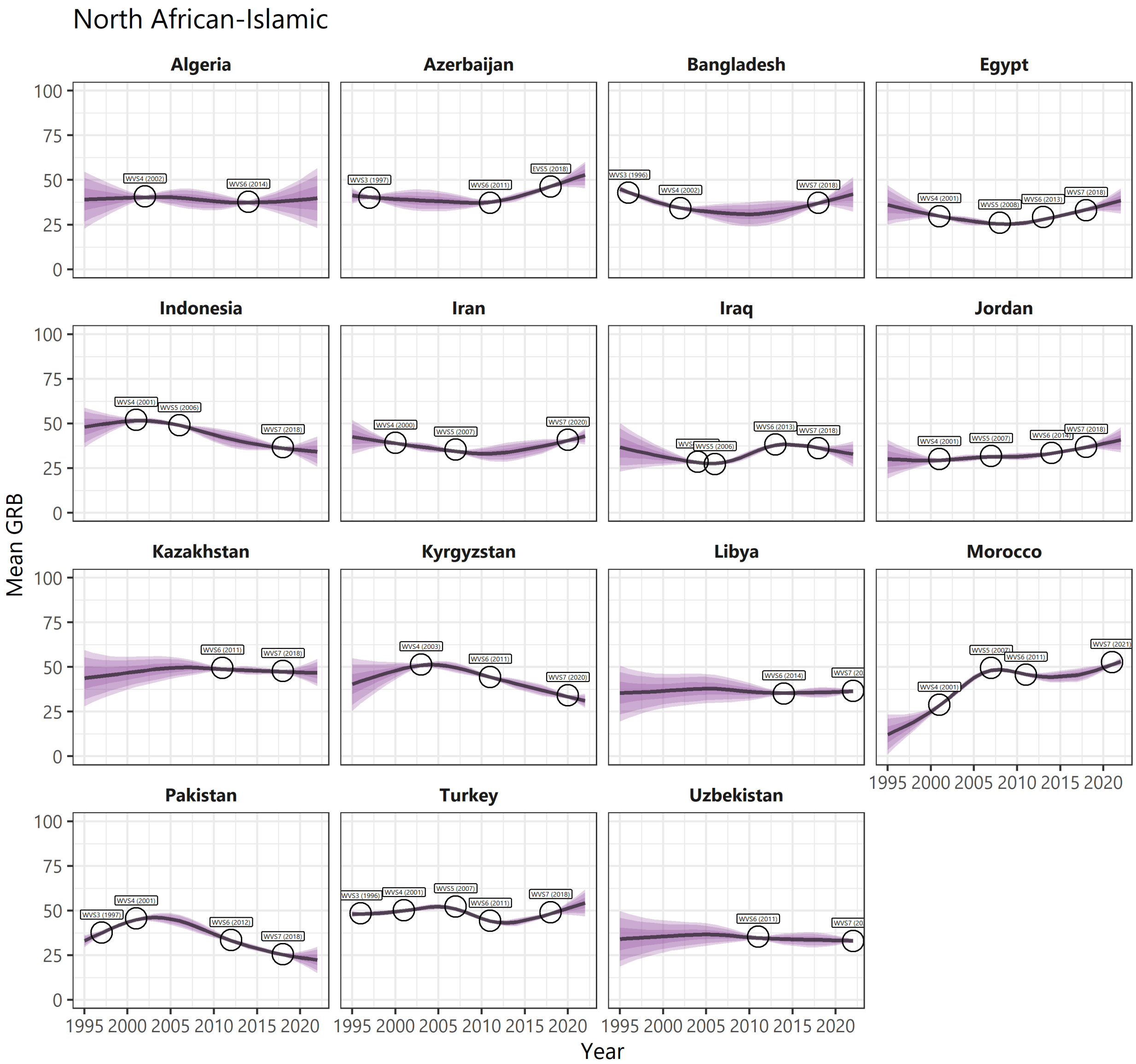

Figure S6: Estimated mean GRB over time of the populations of countries in the cultural zone *North African-Islamic*. Lines are estimates (posterior means) from the HGAM with post-stratification. Pointwise 50%, 80%, and 90% equal-tailed credible intervals are shown in purple. Points are sample means in the survey wave and year noted above each point.

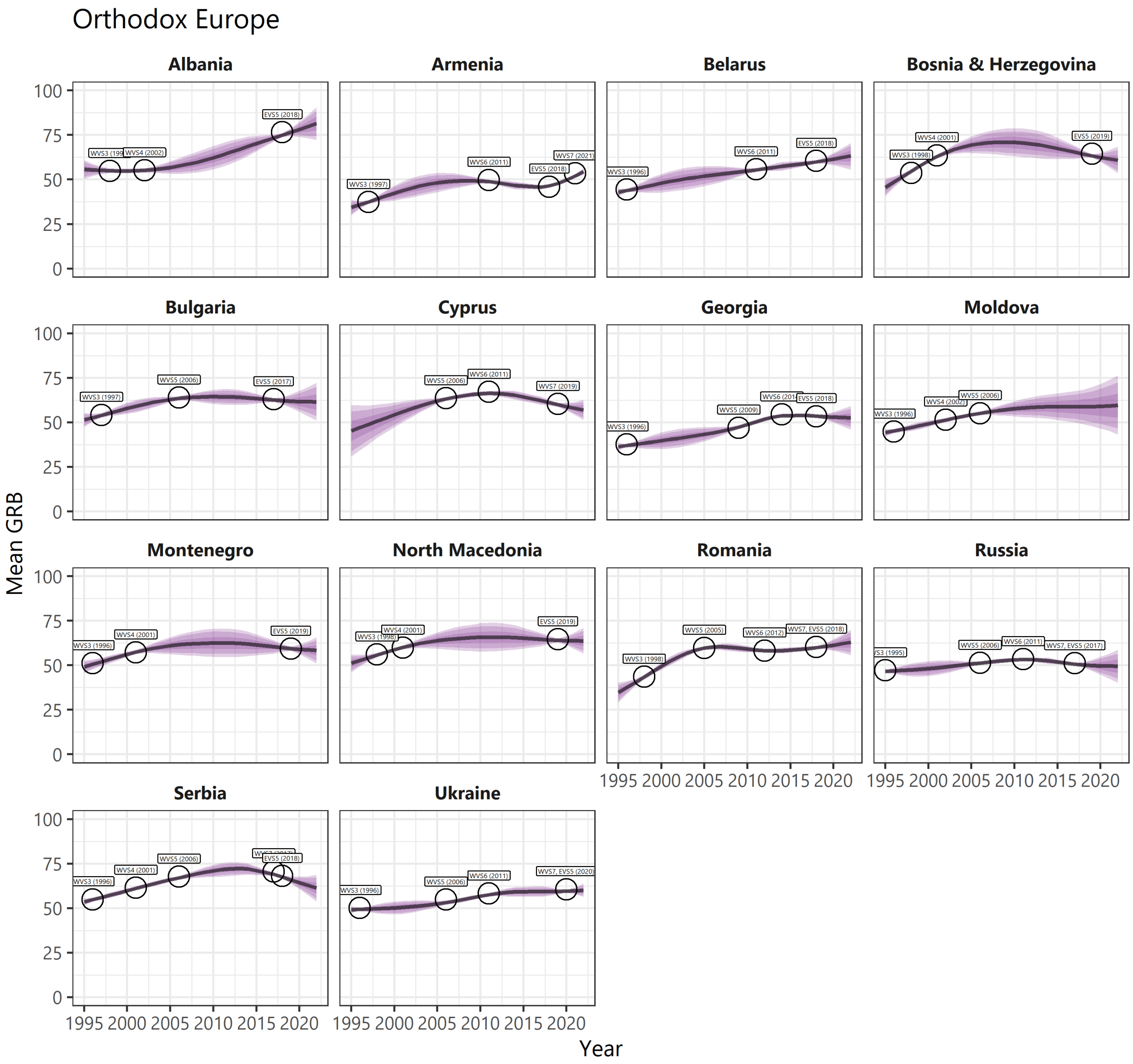

Figure S7: Estimated mean GRB over time of the populations of countries in the cultural zone *Orthodox Europe*. Lines are estimates (posterior means) from the HGAM with post-stratification. Pointwise 50%, 80%, and 90% equal-tailed credible intervals are shown in purple. Points are sample means in the survey wave and year noted above each point.

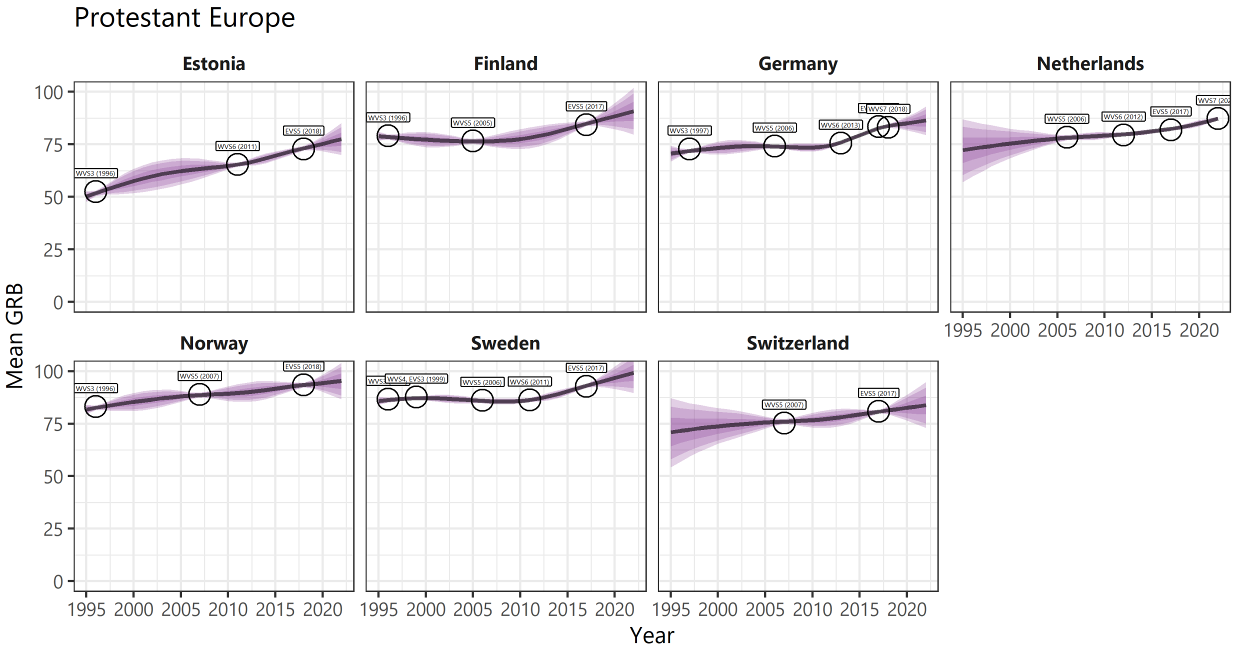

Figure S8: Estimated mean GRB over time of the populations of countries in the cultural zone *Protestant Europe*. Lines are estimates (posterior means) from the HGAM with post-stratification. Pointwise 50%, 80%, and 90% equal-tailed credible intervals are shown in purple. Points are sample means in the survey wave and year noted above each point.

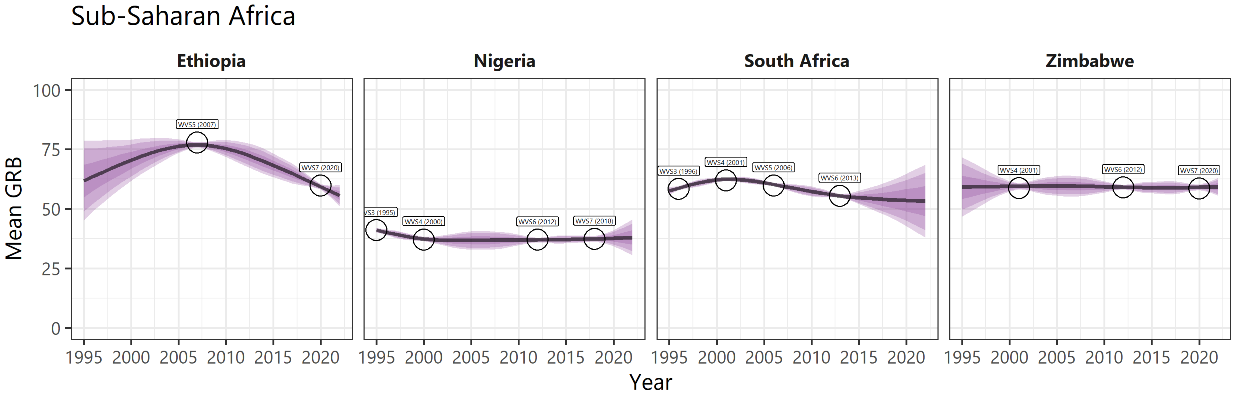

Figure S9: Estimated mean GRB over time of the populations of countries in the cultural zone *Sub-Saharan Africa*. Lines are estimates (posterior means) from the HGAM with post-stratification. Pointwise 50%, 80%, and 90% equal-tailed credible intervals are shown in purple. Points are sample means in the survey wave and year noted above each point.

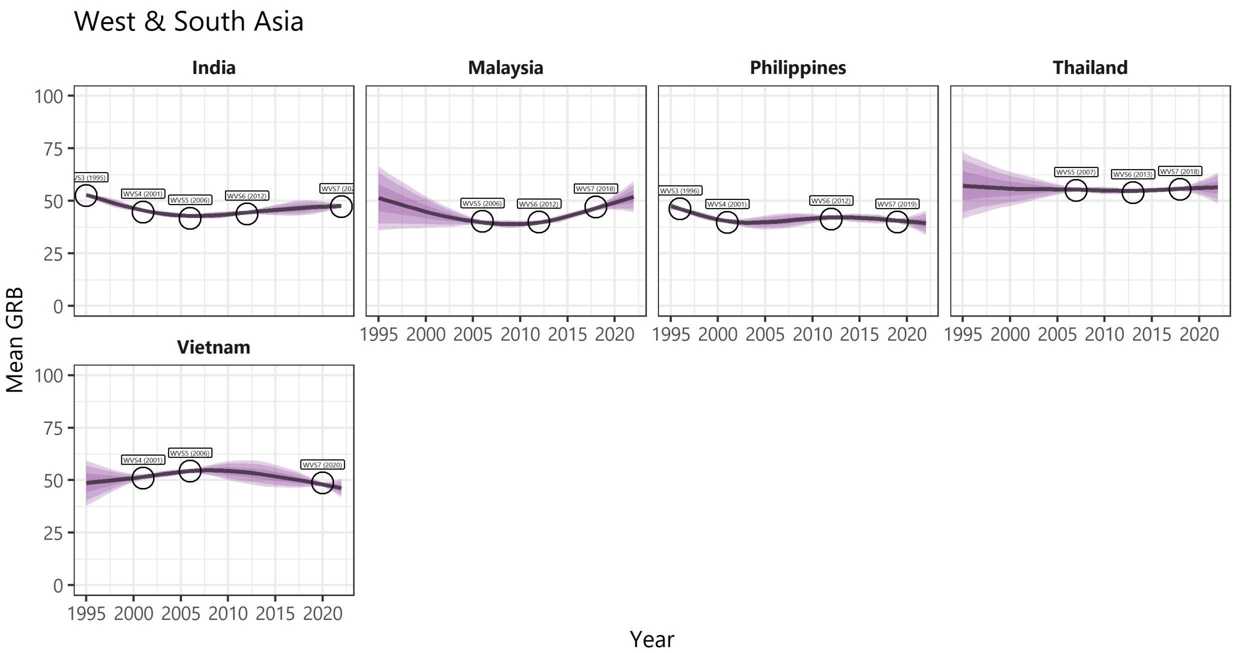

Figure S10: Estimated mean GRB over time of the populations of countries in the cultural zone *West & South Asia*. Lines are estimates (posterior means) from the HGAM with post-stratification. Pointwise 50%, 80%, and 90% equal-tailed credible intervals are shown in purple. Points are sample means in the survey wave and year noted above each point.

S2 Association of GRB and number of children

Figure S11 shows the estimated effects of an individual woman's GRB (horizontal axis) and of the mean GRB among women in the same country (colored lines) on number of children reported in the survey, obtained in the linear mixed-effects model (LMM; see Supplementary Materials S5.3) reported as Model 1 in Table 1 in the main text. The model included only the most recent survey per country, but not older than year 2010. For the figure, effects were back-transformed to the original predictor scale (0-100) and values for the random intercept and slope were set to zero.

The figure allows us to compare the effect of a two-standard-deviation (SD) increase in GRB *within* countries and *between* countries. The between-country effect is markedly larger. Both effect estimates should be interpreted as mere associations, produced by causal processes outside the scope of this study. Results from Models 2 and 3 in Table 1 in the main text demonstrate examples for plausible variables to which some of the covariation could be attributed (e.g., education, religiosity). Note also that the estimated SD in number of children between countries *unexplained* by GRB is comparable in size with the between-country effect of a one SD increase in GRB (see Table 1 in the main text). Hence, there are factors operating on the country level unrelated to GRB that also contribute substantially to variation in (our proxy of) completed cohort fertility.

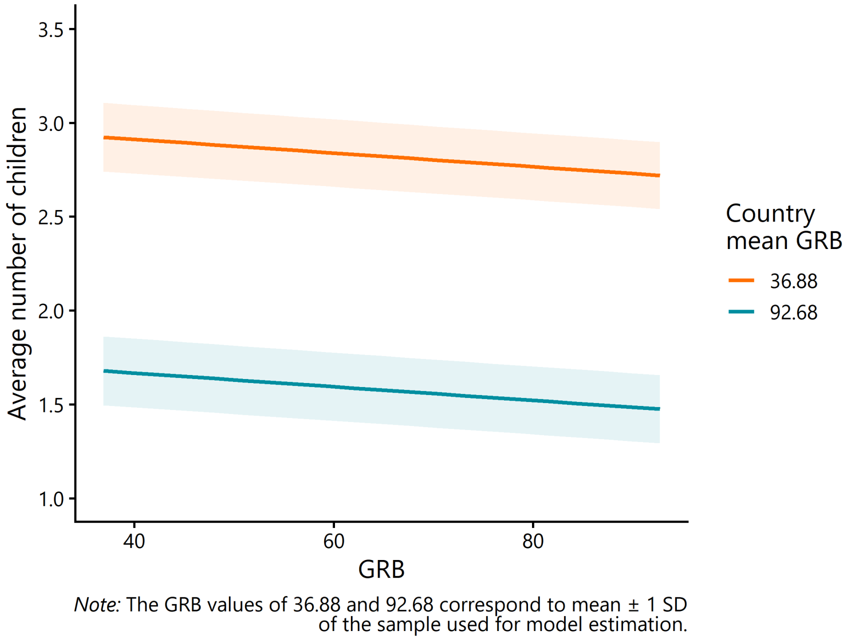

Figure S11: Estimated average number of children as a function of a woman's GRB (horizontal axis) and the sample mean GRB of women in her country (colored lines) with pointwise 90% confidence intervals. Results are based on a linear mixed-effects model that includes random country intercepts and slopes.

S3 Country-specific cumulative age-specific fertility rates

With our Bayesian hierarchical period age-specific fertility rate (ASFR) model, we estimated a country's fertility curve for the recent period (the most recent survey per country, but not older than year 2010; 101 countries). The model assumed that observed numbers of children for women in the population (18-49 years) resulted from a Poisson process, the expectation of which was the cumulative ASFR up until a woman's current age. For more details, see Supplementary Materials S5.4. In Figures S12 to S20, observed numbers of children are plotted against the fitted cumulative ASFR (posterior mean) for the two subpopulations of women with *less egalitarian* GRB (equal to or lower than the global sample median of 66.67) and *more egalitarian* GRB (higher than the global sample median of 66.67) for all countries. This provides an informal way to assess model fit. Another way to read the figures is that the line represents the area under the fertility curve. For the figures, the observed numbers of children were averaged if there was more than one woman sampled for a certain country, subpopulation, and age (point size indicates sample size). The year in parentheses is the year in which the data were collected, i.e., in which the country was last surveyed. For each cultural zone in the sample, one figure is shown.

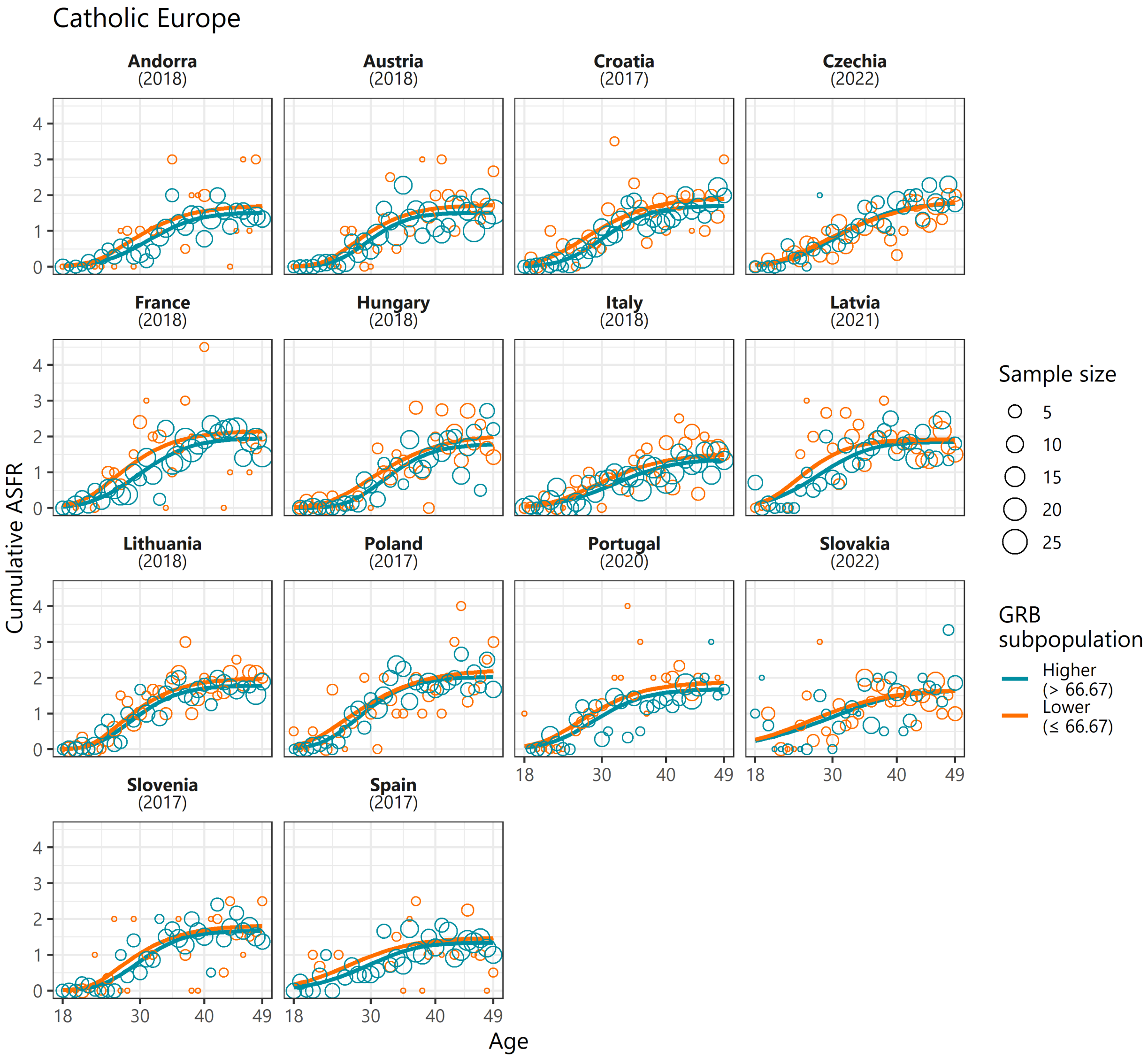

Figure S12: Estimated cumulative age-specific fertility rate (ASFR) of countries in the cultural zone *Catholic Europe*. Lines are fits from the Bayesian hierarchical ASFR model (posterior means). Points are (averages of) observed number of children among women surveyed in the country and year noted above each panel.

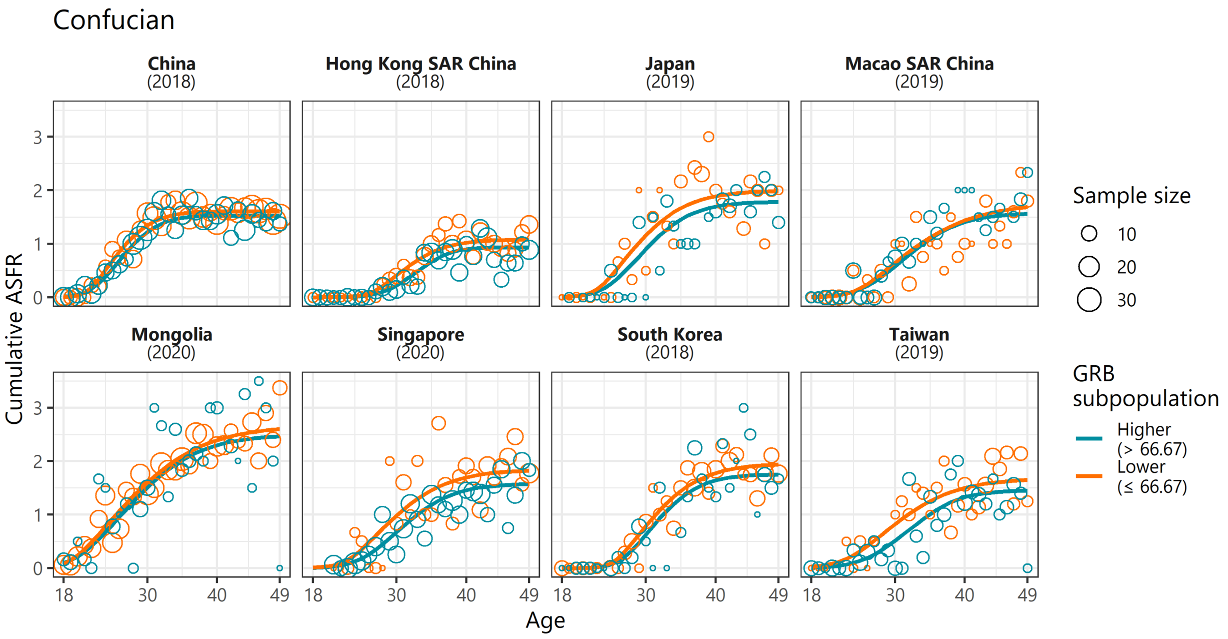

Figure S13: Estimated cumulative age-specific fertility rate (ASFR) of countries in the cultural zone *Confucian*. Lines are fits from the Bayesian hierarchical ASFR model (posterior means). Points are (averages of) observed number of children among women surveyed in the country and year noted above each panel.

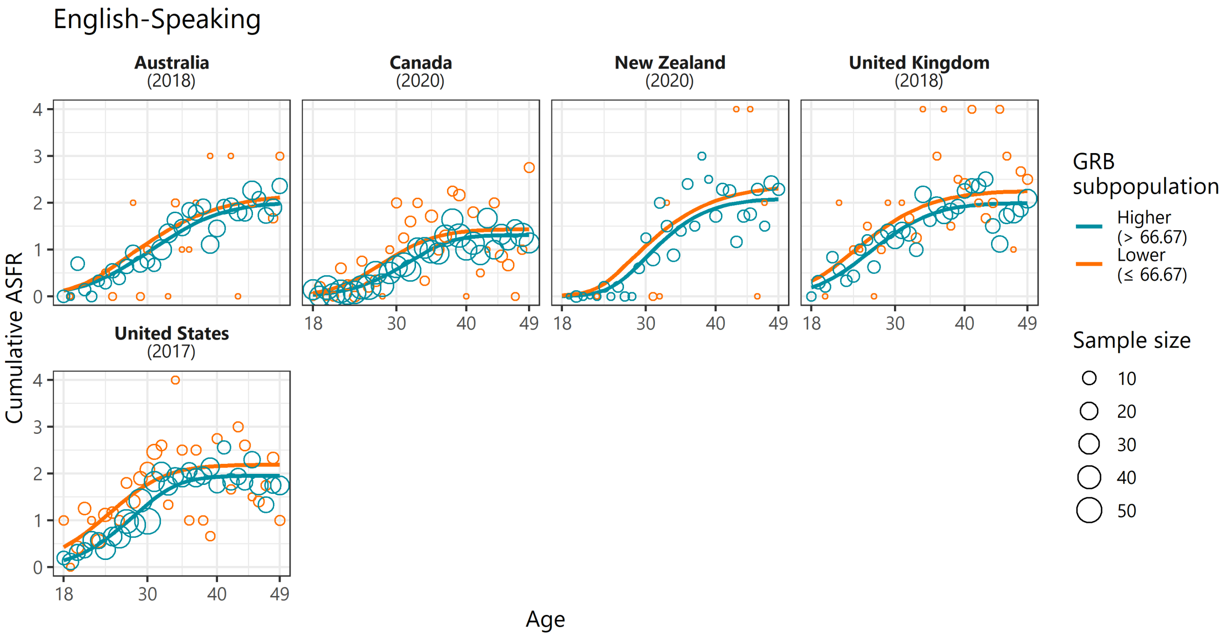

Figure S14: Estimated cumulative age-specific fertility rate (ASFR) of countries in the cultural zone *English-Speaking*. Lines are fits from the Bayesian hierarchical ASFR model (posterior means). Points are (averages of) observed number of children among women surveyed in the country and year noted above each panel.

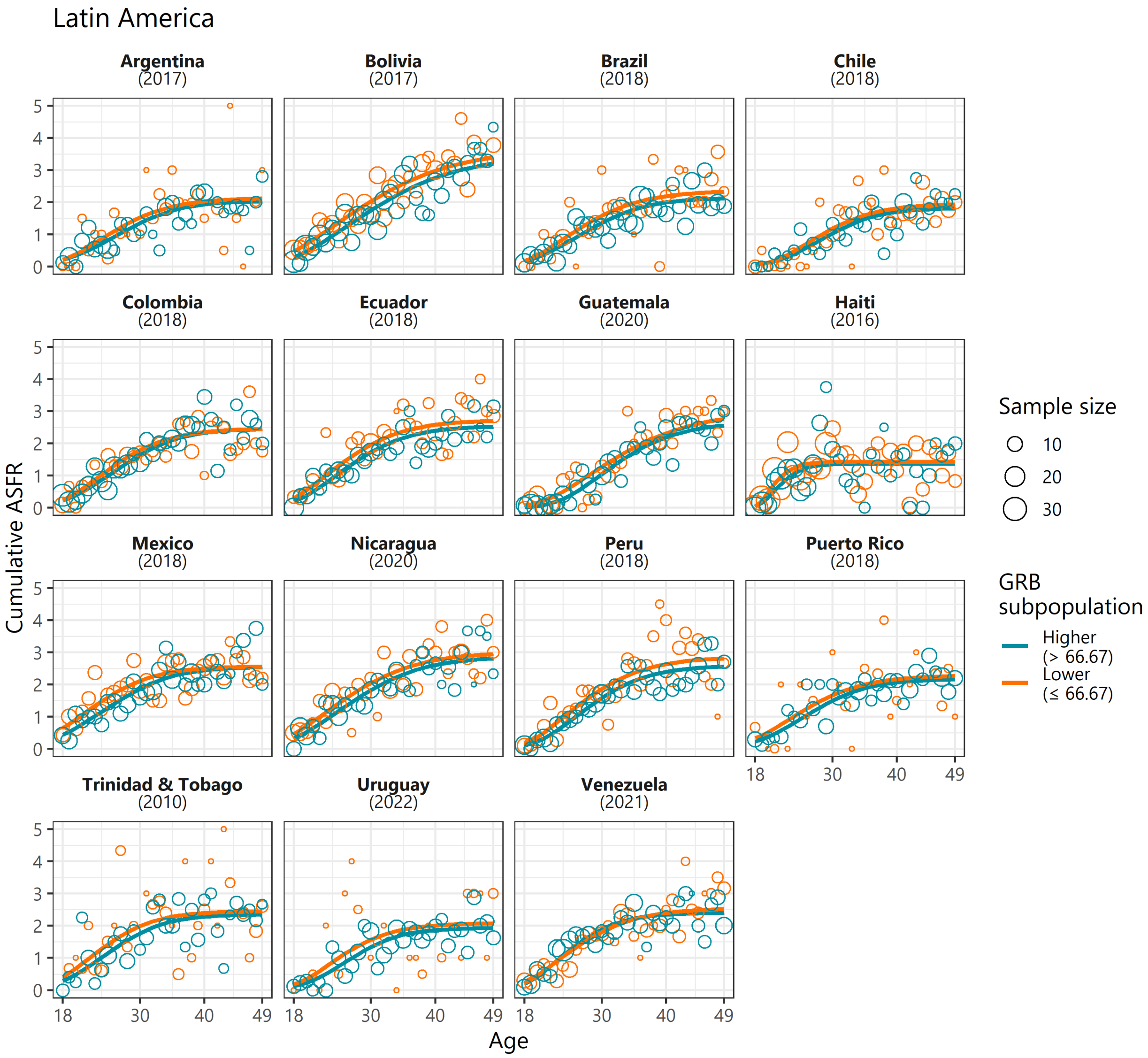

Figure S15: Estimated cumulative age-specific fertility rate (ASFR) of countries in the cultural zone *Latin America*. Lines are fits from the Bayesian hierarchical ASFR model (posterior means). Points are (averages of) observed number of children among women surveyed in the country and year noted above each panel.

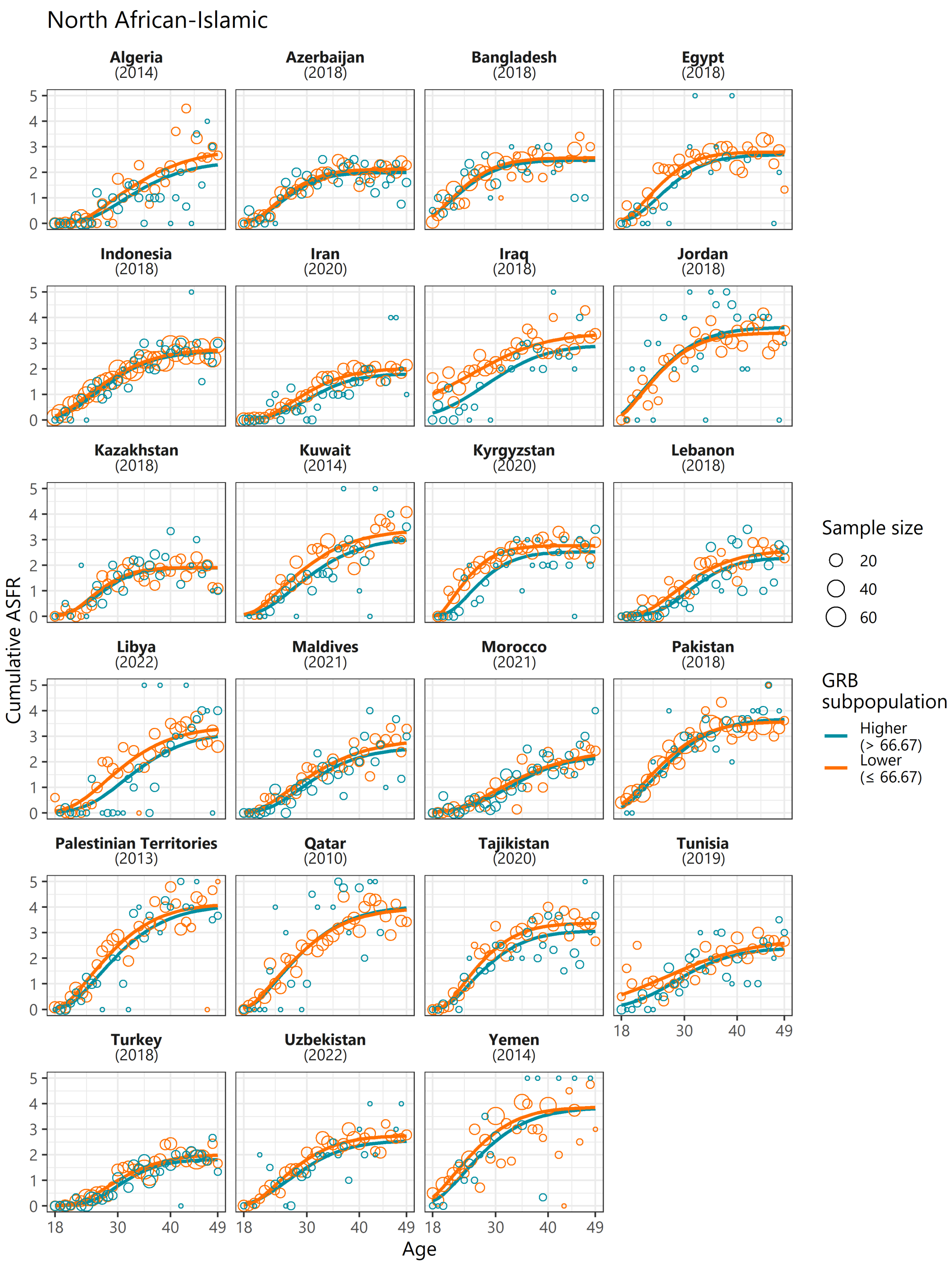

Figure S16: Estimated cumulative age-specific fertility rate (ASFR) of countries in the cultural zone *North African-Islamic*. Lines are fits from the Bayesian hierarchical ASFR model (posterior means). Points are (averages of) observed number of children among women surveyed in the country and year noted above each panel.

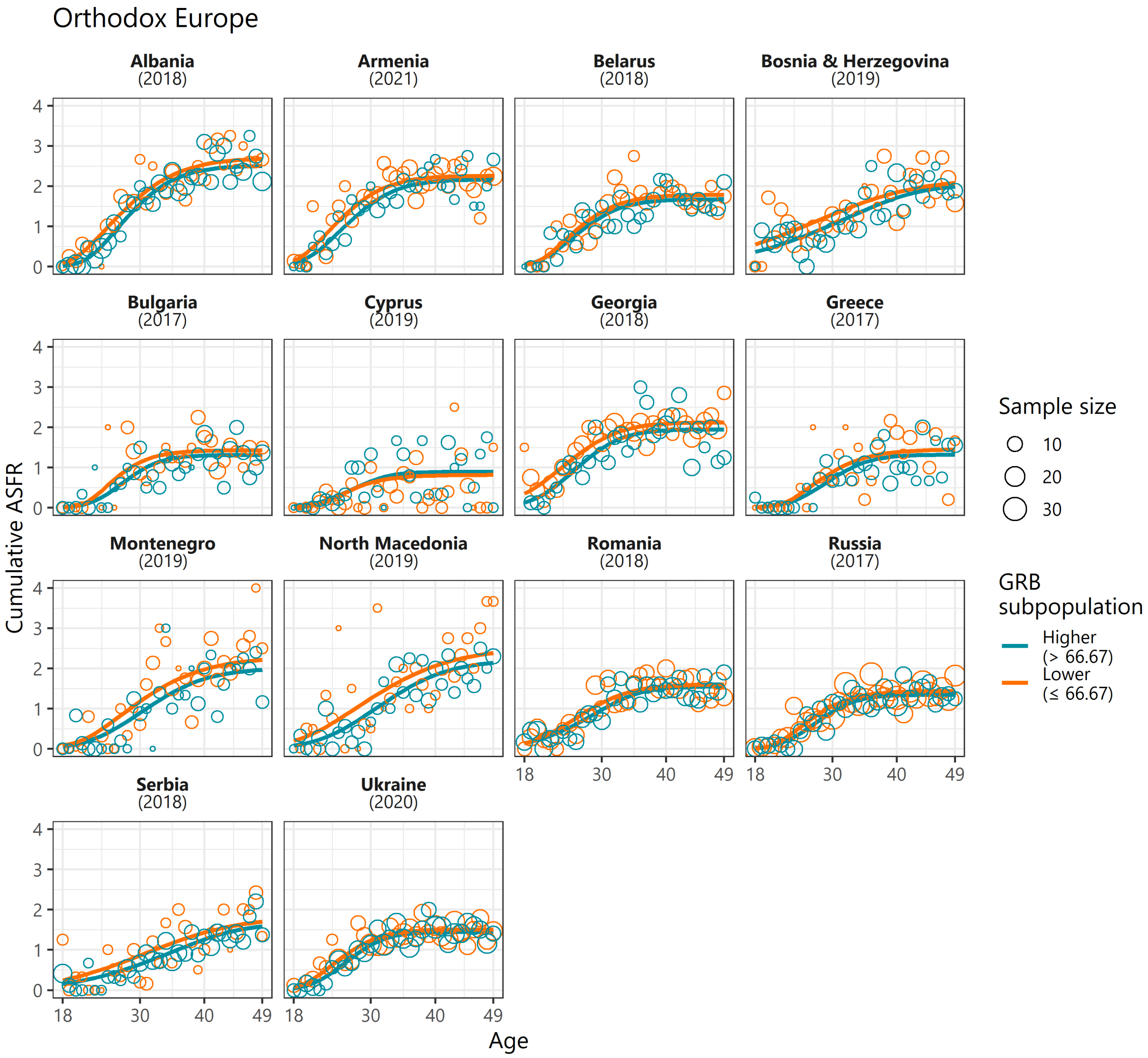

Figure S17: Estimated cumulative age-specific fertility rate (ASFR) of countries in the cultural zone *Orthodox Europe*. Lines are fits from the Bayesian hierarchical ASFR model (posterior means). Points are (averages of) observed number of children among women surveyed in the country and year noted above each panel.

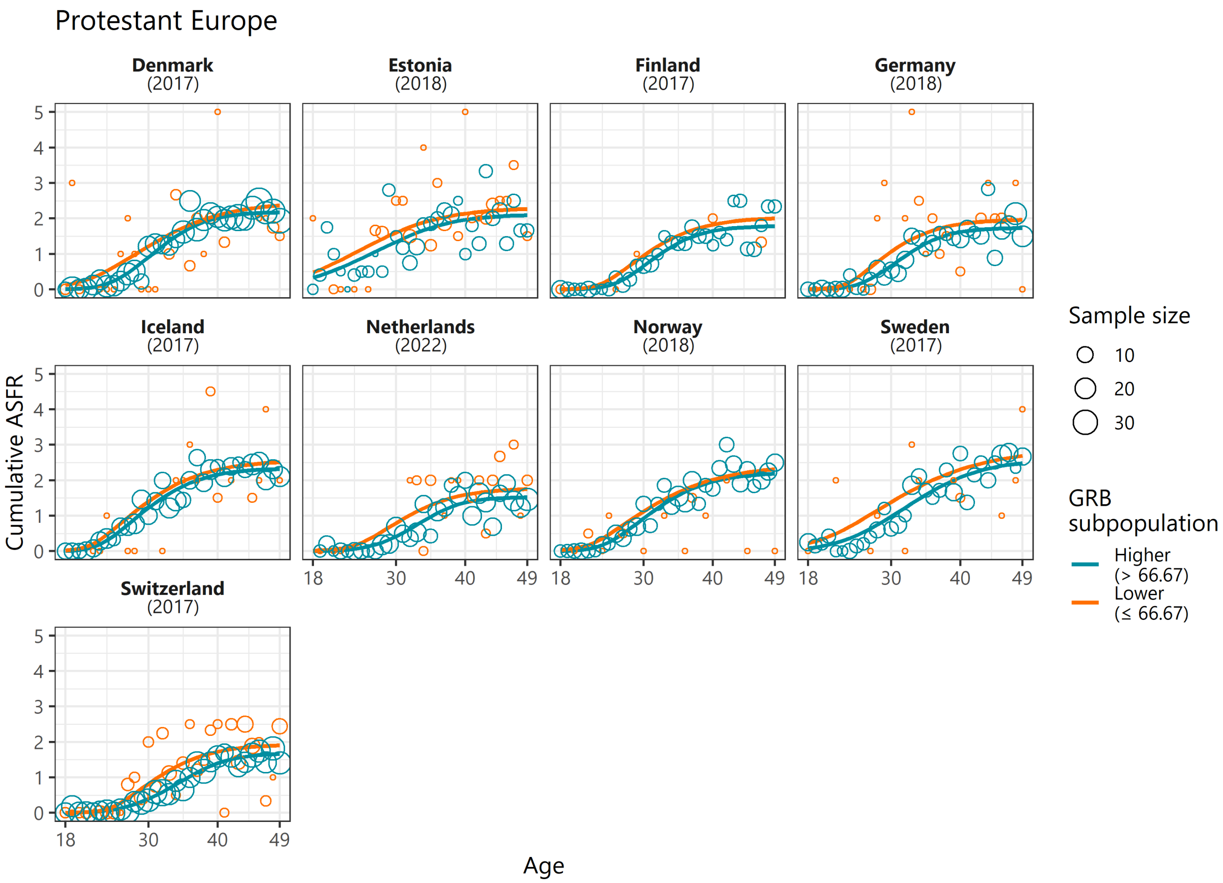

Figure S18: Estimated cumulative age-specific fertility rate (ASFR) of countries in the cultural zone *Protestant Europe*. Lines are fits from the Bayesian hierarchical ASFR model (posterior means). Points are (averages of) observed number of children among women surveyed in the country and year noted above each panel.

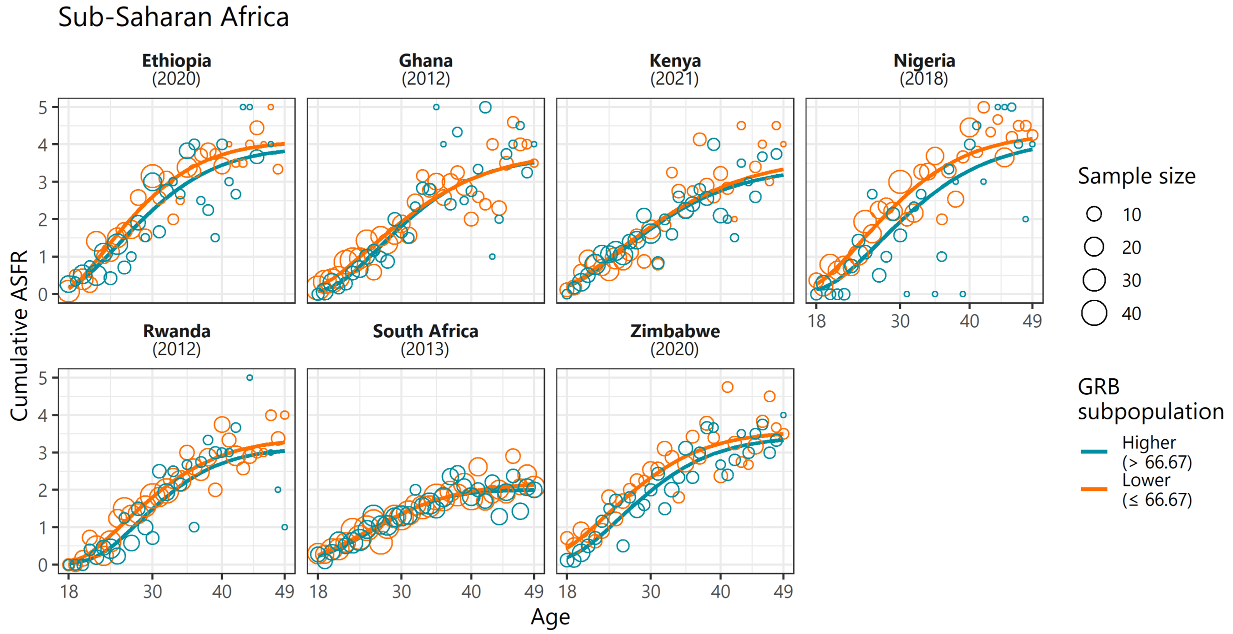

Figure S19: Estimated cumulative age-specific fertility rate (ASFR) of countries in the cultural zone *Sub-Saharan Africa*. Lines are fits from the Bayesian hierarchical ASFR model (posterior means). Points are (averages of) observed number of children among women surveyed in the country and year noted above each panel.

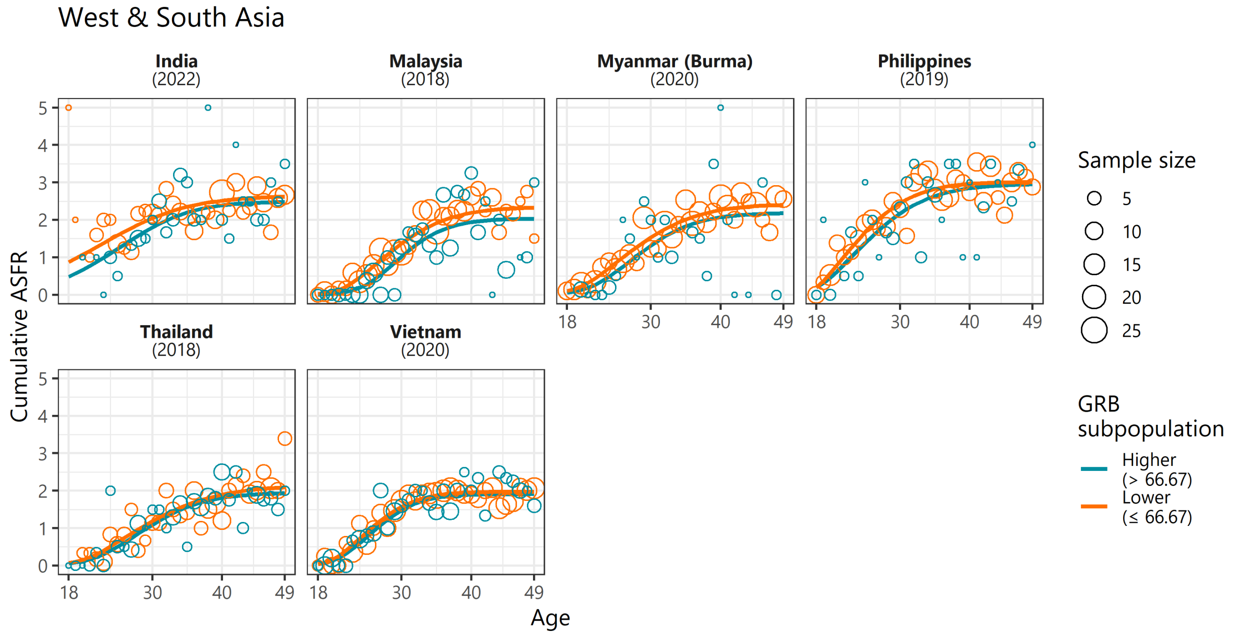

Figure S20: Estimated cumulative age-specific fertility rate (ASFR) of countries in the cultural zone *West & South Asia*. Lines are fits from the Bayesian hierarchical ASFR model (posterior means). Points are (averages of) observed number of children among women surveyed in the country and year noted above each panel.

S4 Country-specific demographic effects on GRB

The HGAM, which was the basis for estimating the country-specific time trends in mean GRB shown in Supplementary Materials S1, also included fixed effects and country-varying random intercepts for the six demographic subgroups that result from interacting the age categories {18-34 years; 35-59 years; 60+ years} with the sex categories {male, female}. In Supplementary Materials S5.2, the model is described in more detail. Figure S21 visualizes between-country variation in terms of the estimated random intercepts, varying around the estimated fixed average effects.[1] In Figure S21, strongly outlying countries are labelled. For example, young South Korean women are estimated to be more gender egalitarian compared to their age-sex group average, whereas old South Korean men are estimated to be less gender egalitarian compared to the their age-sex group average.

The country-varying, time-constant demographic subgroup effects in Figure S21 represent the strata effects used for post-stratification (see Supplementary Materials S5.2).

[1]The estimates of fixed average effects are similar to findings from other survey projects. For example, Charles (2020) reports more gender egalitarian attitudes among women than men in her analysis of Afrobarometer data from 34 African countries (many of which could not be part of our analysis due to lack of data).

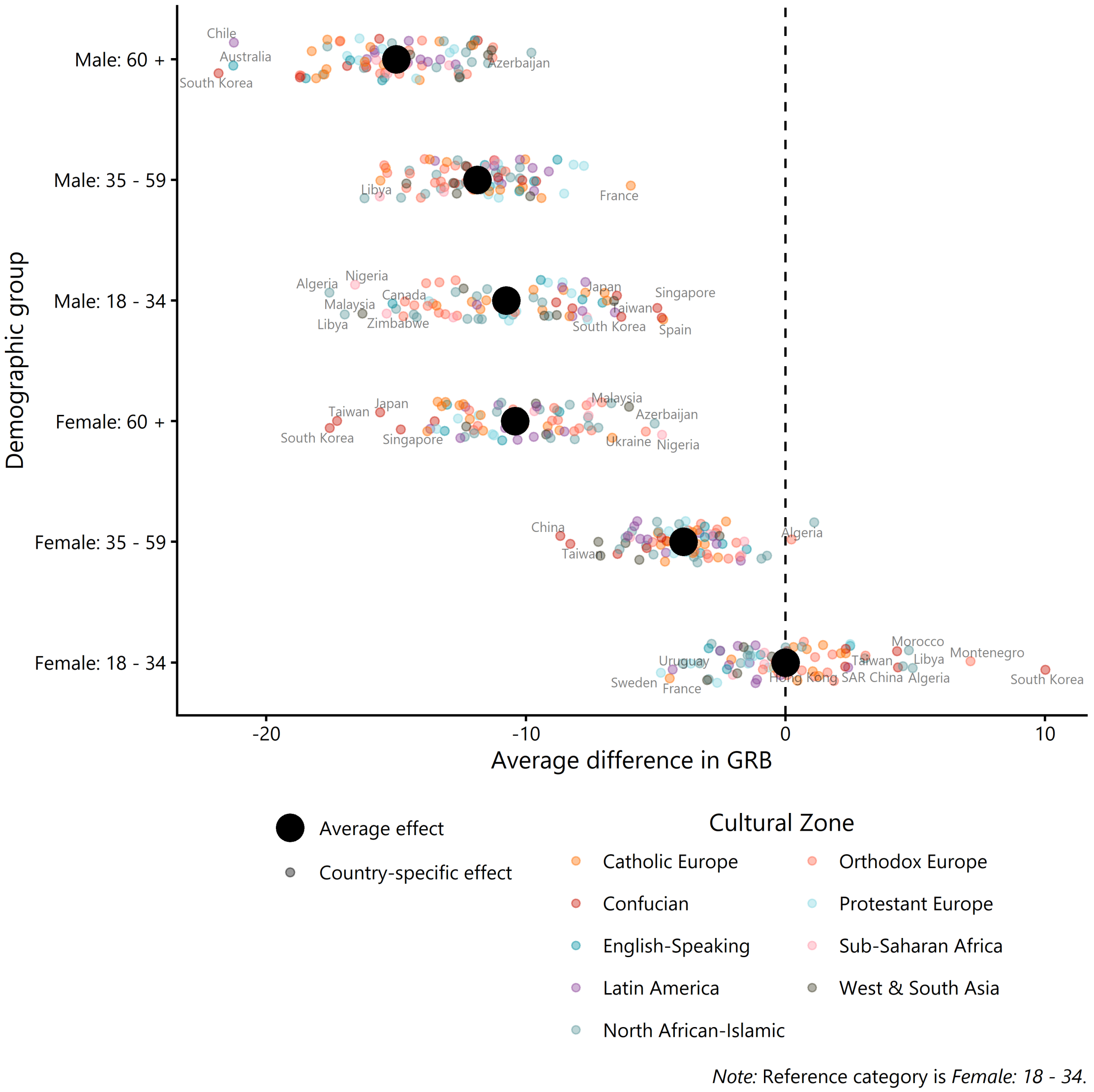

Figure S21: Estimated average and country-specific effects of demographic subgroups on average GRB, relative to the reference category.

S5 Additional methodological details

S5.1 Measurement of GRB

Measure construction

The GRB variable was an index of three component items from the World Values Survey and European Values Study (WEVS). For each item separately, responses were assigned equidistant scores in the range 0-100, with 0 as the lower and 100 as the upper end of the response scale (min-max normalization). Higher values indicated more gender egalitarian beliefs. The arithmetic mean of the three item scores per respondent was taken as the respondent's observed level of GRB. Respondents and surveys were only included in the study if none of the three items was missing.[2] Below, we provide the original item wordings and their scoring[3]

[2]Other survey projects we considered for extending the dataset, like the *Pew Research Global Attitudes & Trends Survey* or the *Afrobarometer*, contained only a single analogous item (women's right to a job) and could hence not be included in our study.

[3]Scores shown here are rounded to two digits, but we used exact fractions in all calculations.

(see also, e.g., Akaliyski, 2023; Welzel, 2013):

- **Item: Education.** For each of the following statements I read out, can you tell me how much you agree with each. Do you agree strongly, agree, disagree, or disagree strongly? A university education is more important for a boy than for a girl.

  Strongly disagree (100) / Disagree (66.67) / Agree (33.33) / Agree strongly (0)

- **Item: Jobs.** Do you agree, disagree or neither agree nor disagree with the following statements? When jobs are scarce, men should have more right to a job than women.

  Disagree (100) / Neither (50) / Agree (0)

- **Item: Politics.** For each of the following statements I read out, can you tell me how much you agree with each. Do you agree strongly, agree, disagree, or disagree strongly? On the whole, men make better political leaders than women do.

  Strongly disagree (100) / Disagree (66.67) / Agree (33.33) / Agree strongly (0)

The distribution of GRB in the global sample used for fitting the HGAM (373,855 respondents from 78 countries surveyed between 1995 and 2022) is visualized in Figure S22. In the same sample, which pools individual responses across countries and survey waves, Cronbach's α for the measure comprised of the three items was estimated as 0.66. Akaliyski (2023) reports results from a principal component analysis of these items on the country level, suggesting the presence of a single component.

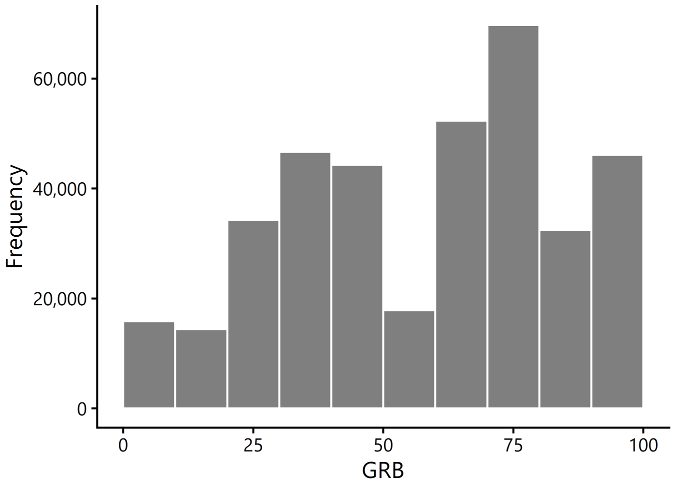

Figure S22: Distribution of GRB in the global sample used for fitting the HGAM.

Measurement properties across cultural zones

We relied on individuals' scores on the GRB measure when estimating our quantities of interest (e.g., global or group-specific trends in mean GRB, or fertility differences among women with different levels of GRB). Accordingly, the assumption that persons from different regions or times understand the same item in an (approximately) equivalent way and assign it the same meaning is important. For this reason, we used a measure that had already been established in cross-cultural research, and we also examined some measurement properties across cultural zones as a way of re-validation.

The construction of the GRB variable (see above) mimicked the three-item *Equality* index that Welzel (2013, p. 71) proposed as a component of the higher-level construct of *Emancipative Values*.[4] Its reliability and

[4]The three-item index is also a pre-computed variable in the World Values Survey dataset (Inglehart et al., 2024).

validity were extensively discussed by Welzel (2013). Previous research has relied on the same measure when analyzing perceptions/norms on gender equality. This includes, for example, research in the fields of cross-cultural psychology (e.g., Akaliyski, 2023; Welzel, 2013), education (e.g., Eriksson et al., 2020), or economics (e.g., Fike, 2024). In an empirical analysis of cross-cultural measurement invariance, Sokolov (2018) indeed concluded for two of the three component items that they might function "cognitively equivalent across different cultures".

We can also examine measurement properties across cultural zones in our global sample used for fitting the HGAM, which pools individual responses across countries and survey waves. Figure S23A presents estimates of standardized factor loadings from a multi-group confirmatory factor analysis (CFA) on the three items using the `lavaan` package (Rosseel, 2012). Model parameters were not constrained across groups, which, in the measurement invariance literature, is also called the *configural model* specification (e.g., Bratt, 2025). Figure S23B presents Cronbach's α estimates. No re-weighting or other adjustments were applied to the sample during analyses.

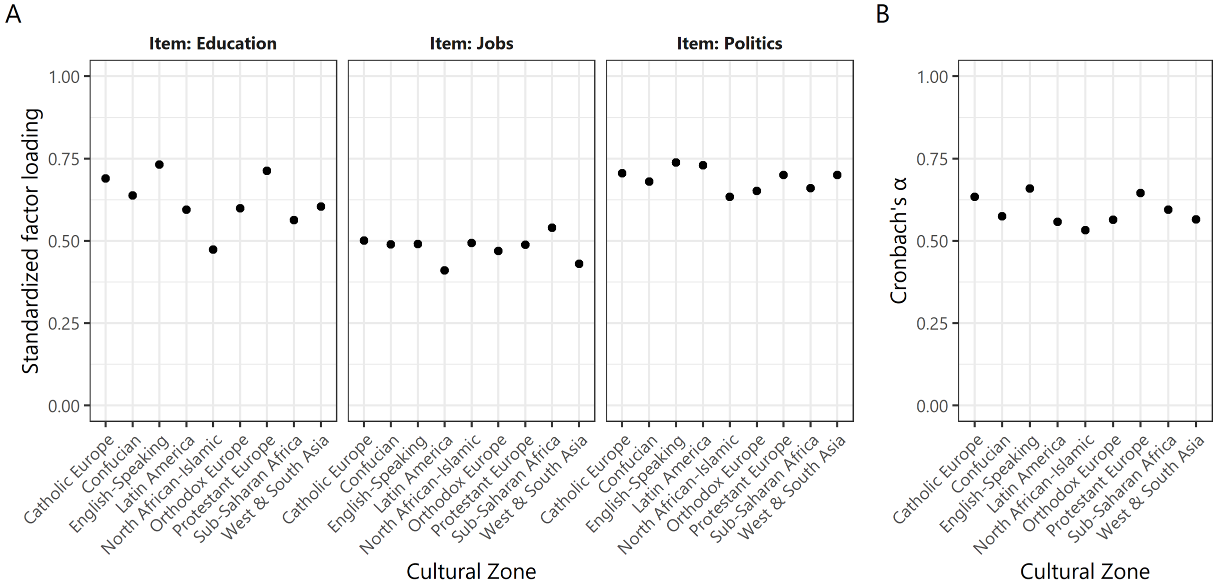

Figure S23: Estimates of measurement properties of the GRB index for all cultural zones.

Standardized factor loadings for all items and cultural zones were ≥ 0.4, a cutoff usually taken to suggest that "the indicator is meaningfully related to a [...] factor" (Brown, 2015, p. 27). The majority of estimates were ≥ 0.6. The item on women's right to a job generally loaded more weakly onto the factor than the other two items. Variation between groups in factor loading was largest for the item on importance of university education for girls. Cronbach's α estimates for all cultural zones were generally similar and ≥ 0.5, but none exceeded 0.7. They indicated a modest internal consistency of the scale.

Figure S23 suggests the presence of some cross-cultural heterogeneity in measurement, but generally estimates were in the same ballpark across cultural zones. Hence, the measure appeared to function similarly across groups.

We provide this coarse exploration of so-called *metric measurement invariance* (e.g., Boer et al., 2018; Sokolov, 2018) as background information for our study. The value of strict measurement invariance testing via the latent variable approach of CFA has been debated for cross-cultural research (e.g., Bratt, 2025; Kusano et al., 2025; Welzel et al., 2023; Welzel & Inglehart, 2016; Zhirkov & Welzel, 2024). This concerns in particular constructs that are to some degree intended to be *formative*, i.e., that are designed

to emerge from the combination of partially complementary (rather than necessarily correlated) items (Welzel et al., 2023; Welzel & Inglehart, 2016). As an alternative means of validation of measurement, the *nomological* approach considers how the measure is empirically associated with external variables that it should theoretically be related to (Kusano et al., 2025; Minkov et al., 2024; Welzel et al., 2023). For our three-item measure, for example, high country-level correlations with a democracy measure focused on citizen and human rights (Sokolov, 2018; Welzel, 2013), a measure of economic freedom (Fike, 2024), or a measure of reproductive freedom values (Akaliyski, 2023) have been reported.

S5.2 The HGAM and post-stratification

General approach

To estimate a smooth effect of time on average GRB per country, at the heart of all our longitudinal analyses was a HGAM (Pedersen et al., 2019) fit with the `mgcv` package v1.9-4 (Wood, 2017) in `R` v4.5.2 (R Core Team, 2025), followed by representativeness adjustments via post-stratification. The approach helps in the face of typical challenges when analyzing panels of survey waves where a smooth continuous time trend in public opinion is of ultimate interest, but survey waves are scattered across years and countries in an unbalanced fashion, and might exhibit sampling bias to different degrees (Kołczyńska et al., 2024). As an example, Fike (2024) analyzed country-level trends in our measure of GRB (which she called "Gender Social Norms Index") and noted the challenges with differential timing and coverage of surveys, making it "difficult to determine how norms have changed over time" (p. 14) based on the raw data alone.

We set the model up in a way that, when no survey sample was available for a country in a given year, information on the average level of GRB was borrowed between neighboring countries in cultural space, as well as within-countries across time, associated with an increase in uncertainty reflected in estimated variances.[5] Further, by relying on external, annual census data for post-stratification, changes in adult population composition that would affect mean GRB (e.g., women typically have more egalitarian views on gender-related topics, see Figure S21) were also captured – even when no new survey data were collected in that year.

Our HGAM of the "GS" type (see nomenclature from Pedersen et al., 2019) has the advantage of being very flexible and allowing for any shape of the individual (here, country-specific) mean GRB trajectories over time. In essence, "fitted curves interpolate the data, but there is a high level of uncertainty about the true trajectory between the data points" (Ogden, 2024, p. 2). This is seen in the credible intervals in Figure S1, which widen substantially in time periods with no survey coverage, but become extremely narrow in surveyed years. Note that this uncertainty is propagated through our whole analysis. A weakness of the model is that it does not exploit structural similarities in the shape of the time effect between countries (Ogden, 2024).

We believe that the method (HGAM with post-stratification) provided plausible country-specific estimates of the trend in population mean GRB, together with a principled quantification of statistical uncertainty (Figure S1). In particular, the method is a potential alternative to more crude approaches sometimes applied to longitudinal, multi-country survey datasets. This might include last-observed-value-carried-forward imputation, linear interpolation, or a reduction of the sample to only countries with very frequent survey coverage (which hardly leads to datasets allowing conclusions about global trends). We view our method as extending the toolbox to estimate long-term attitudinal change in the global population from WEVS and similar survey datasets. For other approaches – some more, some less model-based – see, for example, Anderson et al. (2021) or Foa et al. (2022).[6]

To avoid excessive extrapolation, countries were only included in the sample of the longitudinal analysis when they had ≥ 2 national WEVS surveys between 1995 and 2022, and these spanned at least a quarter of the observation period (i.e., they were at least 7 years apart). Since two of the three items were introduced into questionnaires only with wave 3 of the World Values Survey (WVS), no older data could be used. The 78 included countries[7] made up 86.1% of the world's adult (18+ years) population in 2022. The resulting

[5]Note that, compared to some previous related work (Claassen, 2019; Kołczyńska et al., 2024; Kołczyńska & Bürkner, 2024; Solt, 2020), we did not specify a latent trait model or item response theory model, but a simple Gaussian model. The former are particularly helpful when item phrasing and response options are also heterogeneous across surveys in a relevant way, which was not the case in our dataset.

[6]In an early phase of our study, we also analyzed the global and cultural zone-specific trajectories of population mean GRB with a method closely following Foa et al. (2022): We restricted the dataset to countries with at least a 15-year span of available data, always entered the mean GRB estimate of the most recent available survey in each year for each country, and computed annual global averages weighted by national population sizes. Results were qualitatively very similar, but they were based on a smaller set of countries (i.e., smaller share of the global population), they exhibited some unrealistic "jumps" in the longitudinal profiles, and did not provide a straightforward way to inspect statistical uncertainty.

[7]For simplicity, "countries" refers to all surveyed entities, including territories and other political units.

sample for model estimation is described in Table S1. It contained n = 373,855 respondents with non-missing entries for all variables, originating from 78 countries in 9 cultural zones, belonging to 6 age-sex groups and having been surveyed in 270 national surveys (counting only one survey per year in the rare cases where multiple occurred) in 26 distinct years.[8]

Table S1: Sample sizes per country and survey year in the sample used for fitting the HGAM.

Country	Year (sample size)	Country	Year (sample size)
Albania	1998 (840); 2002 (880); 2018 (1,375)	Libya	2014 (2,038); 2022 (1,165)
Algeria	2002 (1,170); 2014 (1,121)	Lithuania	1997 (809); 2018 (1,231)
Andorra	2005 (980); 2018 (989)	Malaysia	2006 (1,062); 2012 (1,300); 2018 (1,313)
Argentina	1995 (957); 1999 (1,113); 2006 (865); 2013 (955); 2017 (941)	Mexico	1996 (1,271); 2000 (1,402); 2005 (1,468); 2012 (1,970); 2018 (1,721)
Armenia	1997 (1,609); 2011 (1,053); 2018 (1,451); 2021 (1,159)	Moldova	1996 (889); 2002 (860); 2006 (1,006)
Australia	1995 (1,825); 2005 (1,369); 2012 (1,415); 2018 (1,758)	Montenegro	1996 (189); 2001 (850); 2019 (942)
Azerbaijan	1997 (1,724); 2011 (999); 2018 (1,657)	Morocco	2001 (991); 2007 (1,105); 2011 (935); 2021 (1,200)
Bangladesh	1996 (1,313); 2002 (1,432); 2018 (1,122)	Netherlands	2006 (911); 2012 (1,622); 2017 (2,286); 2022 (1,899)
Belarus	1996 (1,796); 2011 (1,432); 2018 (1,441)	New Zealand	1998 (945); 2004 (771); 2011 (717); 2020 (892)
Bosnia & Herzegovina	1998 (1,131); 2001 (1,152); 2019 (1,649)	Nigeria	1995 (1,802); 2000 (1,931); 2012 (1,759); 2018 (1,206)
Brazil	1997 (1,109); 2006 (1,476); 2014 (1,408); 2018 (1,557)	North Macedonia	1998 (871); 2001 (987); 2019 (1,018)
Bulgaria	1997 (792); 2006 (881); 2017 (1,378)	Norway	1996 (1,111); 2007 (1,018); 2018 (1,110)
Canada	2000 (1,844); 2006 (2,017); 2020 (4,018)	Pakistan	1997 (733); 2001 (1,823); 2012 (1,139); 2018 (1,919)
Chile	1996 (920); 2000 (1,128); 2006 (946); 2012 (922); 2018 (941)	Peru	1996 (1,081); 2001 (1,402); 2006 (1,419); 2012 (1,112); 2018 (1,350)
China	1995 (1,323); 2001 (881); 2007 (1,519); 2013 (1,973); 2018 (3,023)	Philippines	1996 (1,170); 2001 (1,185); 2012 (1,199); 2019 (1,195)
Colombia	1998 (2,898); 2012 (1,451); 2018 (1,520)	Poland	1997 (844); 2005 (830); 2012 (819); 2017 (1,199)
Croatia	1996 (1,112); 2017 (1,433)	Puerto Rico	1995 (1,075); 2001 (678); 2018 (1,105)
Cyprus	2006 (1,043); 2011 (969); 2019 (922)	Romania	1998 (982); 2005 (1,490); 2012 (1,301); 2018 (2,500)
Czechia	1998 (1,025); 2017 (1,551); 2022 (1,158)	Russia	1995 (1,726); 2006 (1,725); 2011 (2,246); 2017 (3,309)
Egypt	2001 (2,880); 2008 (3,039); 2013 (1,523); 2018 (1,166)	Serbia	1996 (1,123); 2001 (987); 2006 (1,137); 2017 (986); 2018 (1,373)
Estonia	1996 (949); 2011 (1,431); 2018 (1,184)	Singapore	2002 (1,266); 2012 (1,938); 2020 (1,981)
Ethiopia	2007 (1,380); 2020 (1,202)	Slovakia	1998 (982); 2017 (1,346); 2022 (1,163)
Finland	1996 (892); 2005 (982); 2017 (1,130)	Slovenia	1995 (896); 2005 (953); 2011 (994); 2017 (1,022)
France	2006 (967); 2018 (1,763)	South Africa	1996 (2,415); 2001 (2,547); 2006 (2,629); 2013 (3,253)
Georgia	1996 (1,809); 2009 (1,355); 2014 (1,137); 2018 (2,089)	South Korea	1996 (1,236); 2001 (1,060); 2005 (1,197); 2010 (1,176); 2018 (1,245)
Germany	1997 (1,883); 2006 (1,864); 2013 (1,975); 2017 (2,070); 2018 (1,484)	Spain	1995 (1,079); 2000 (1,091); 2007 (1,119); 2011 (1,109); 2017 (1,169)
Guatemala	2004 (948); 2020 (1,191)	Sweden	1996 (905); 1999 (1,904); 2006 (979); 2011 (1,154); 2017 (1,169)
Hong Kong SAR China	2005 (1,172); 2014 (976); 2018 (2,040)	Switzerland	2007 (1,189); 2017 (3,091)
Hungary	1998 (601); 2009 (938); 2018 (1,417)	Taiwan	1998 (716); 2006 (1,220); 2012 (1,132); 2019 (1,190)
India	1995 (1,632); 2001 (1,584); 2006 (1,560); 2012 (3,383); 2022 (1,300)	Thailand	2007 (1,510); 2013 (1,157); 2018 (1,333)
Indonesia	2001 (917); 2006 (1,848); 2018 (3,180)	Turkey	1996 (1,762); 2001 (3,248); 2007 (1,262); 2011 (1,540); 2018 (2,295)
Iran	2000 (1,999); 2007 (2,582); 2020 (1,455)	Ukraine	1996 (2,056); 2006 (874); 2011 (1,419); 2020 (2,526)
Iraq	2004 (2,175); 2006 (2,517); 2013 (1,168); 2018 (1,180)	United Kingdom	2005 (886); 2018 (1,729); 2022 (2,368)
Italy	2005 (915); 2018 (2,170)	United States	1995 (1,376); 1999 (1,122); 2006 (1,223); 2011 (2,192); 2017 (2,573)
Japan	1995 (665); 2000 (832); 2005 (636); 2010 (1,331); 2019 (961)	Uruguay	1996 (902); 2006 (871); 2011 (868); 2022 (816)
Jordan	2001 (1,195); 2007 (1,170); 2014 (1,181); 2018 (1,184)	Uzbekistan	2011 (1,439); 2022 (1,167)
Kazakhstan	2011 (1,500); 2018 (1,193)	Venezuela	1996 (1,002); 2000 (1,144); 2021 (1,190)
Kyrgyzstan	2003 (1,018); 2011 (1,489); 2020 (1,160)	Vietnam	2001 (897); 2006 (1,440); 2020 (1,200)
Latvia	1996 (1,073); 2021 (1,204)	Zimbabwe	2001 (931); 2012 (1,500); 2020 (1,182)

In a sensitivity analysis, we applied stricter sample inclusion criteria: A country was only part of the longitudinal analysis if it had ≥ 3 national surveys that jointly covered at least half of the observation period (i.e., 14 years). Results of the sensitivity analysis are presented in Supplementary Materials S6.

[8]For wave 7 of the WVS (official time frame 2017–2022), we set the observed year for India to 2022, although India's national survey was completed only in July 2023. India was the only country in the dataset surveyed after 2022. This manipulation allowed us to include recent data from a large share of the adult world population without having to estimate a (highly uncertain) 2023 population mean for all 77 other countries.

Model definition and fitting

Denote by y_i the GRB of person i, by $j[i]$ the country of the ith person, by $c[j]$ the cultural zone[9] of the jth country, and by $t[i]$ the year of the response of the ith person. We define further $\beta_{k[i]}$ as fixed effect of the demographic (age-sex) group k to which respondent i belongs, and $\alpha_{k[i]}^{j[i]}$ as random country-specific effect for the demographic (age-sex) group k to which respondent i belongs (i.e., the country-specific deviation from the average effect of being in the demographic group k). The 6 categories for demographic groups resulted from interacting the age categories {18-34 years; 35-59 years; 60+ years} with the sex categories {male, female}.[10] One category's effect (reference category: female, 18-34 years) gets absorbed into the intercept term β_0. The model is then defined as

$$y_i = \beta_0 + \beta_{k[i]} + \alpha_{k[i]}^{j[i]} + f_{j[i]}(t[i]) + f_{c[j[i]]}(t[i]) + \epsilon_i,$$

for $i = 1, \dots, n$, where $f_j(t)$ and $f_c(t)$ are unknown country- and cultural zone-specific smooth functions of time, respectively. Gaussian errors $\epsilon_i \sim \mathcal{N}(0, \sigma^2)$ and $\alpha_k^j \sim \mathcal{N}(0, \tau^2)$ are assumed. Estimation of functions $f_j(t)$ and $f_c(t)$ is based on the factor-smoother interaction spline basis (`"fs"` basis in `mgcv`). These are also called "random wiggly curves" (Wood, 2017, sec. 7.7.4), as, similar to conventional random intercepts, they are assumed as random functions and are penalized for deviations from a population average curve. Our approach corresponds to the model type "GS" ("group−level trends, similar smoothness, shared trend") in the nomenclature of Pedersen et al. (2019): All country-specific splines share a common penalty parameter, inducing similar smoothness. Further, they are shrunk towards the average GRB at time t in their cultural zone, i.e., they share a trend with countries in their cultural proximity (this is because of the presence of the term $f_c(t)$ in the model). All cultural zone-specific splines share a common penalty parameter as well, also inducing similar smoothness among cultural zones. Yet, they are not shrunk towards the global (i.e., worldwide) average trend in GRB. Thus, this corresponds to a model of type "G" ("group−level trends, similar smoothness, no shared trend"). A function for the global trend was omitted from the model because survey samples from WEIRD countries were over-represented, which would induce shrinkage of the GRB trend of all countries of the world mainly towards the trend among such countries. We hence avoided the assumption of a shared trend among all countries worldwide. The spline basis includes group-specific intercepts, i.e., random time-independent country and cultural zone effects, which were hence not added explicitly to the model specification above. Most important for our further analysis was the conditional expectation of GRB for a given demographic group k in a country j at time t, which we denote by

$$\mu_k^j(t) = \beta_0 + \beta_k + \alpha_k^j + f_j(t) + f_{c[j]}(t).$$

The model was estimated via `fREML` using the `bam()` function, with settings `k = 10` (basis size/rank) and `m = 2` (i.e., penalty on second derivative) for the two smoothers. Increasing `k` to higher values (15, 20), i.e., allowing more complex models, did not lead to notably different estimated smooth functions or effective degrees of freedom. GRB and year were standardized to have mean zero and SD one before fitting, but effects were back-transformed to the original scale afterwards for figures and further analysis. REML estimates $\widehat{\beta}_k + \widehat{\alpha}_k^j$ are shown in Figure S21.

After fitting, we obtained 2,000 posterior draws of $\mu_k^j(t)$ for all demographic groups and countries in the sample and for all years in the observation period (1995 to 2022), based on the multivariate normal approximation of the joint distribution of the model parameters. This was done with the helper function `fitted_samples(..., method = "gaussian", mvn_method = "mgcv")` from the `gratia` package v0.11.1

[9]Adapted from Welzel (2013) and shown in Figure S1. See also the Inglehart-Welzel World Cultural Map 2023 at `https://www.worldvaluessurvey.org/WVSContents.jsp?CMSID=findings` (accessed January 2025).

[10]See Ghitza & Gelman (2013) for the advantages of deeply interacted and geographically varying subgroup-effects in multilevel models used with post-stratification.

(Simpson, 2024). The draws allow post-stratified inference on longitudinal trends among the global population (research question RQ1) and among populations of different cultural (cultural zones) and socioeconomic (Human Development Index [HDI] categories) contexts (RQ2)[11], as outlined below.

Post-stratification

Parameter estimates and external data on (sub)population sizes were used for the "multilevel regression and poststratification" approach (Gelman & Little, 1997; Ghitza & Gelman, 2013; Lax & Phillips, 2009; Park et al., 2004). This technique allowed us to adjust for potentially unrepresentative survey samples (with respect to age and sex) and to account for changes in national population composition even in years where no survey wave took place. The estimand of interest was the mean GRB of a country's adult population in a given year (i.e., marginalized over the national composition of demographic groups).

We combined estimates from a HGAM with census information on demographic groups across countries and years (see Kołczyńska et al., 2024). We post-stratified on age-sex groups by year and country, relying on UN's World Population Prospects 2024 data on current and historical annual national population sizes by age and sex (age bins defined equivalently to the WEVS sample). The data were downloaded from the UN's Department of Economic and Social Affairs Population Division (data portal at `https://population.un.org/wpp/`; accessed January 2025) and provided the population sizes $N_k^j(t)$. The posterior distribution of the post-stratified population mean GRB at time t for each country j was then computed as

$$\mu^j(t) = \frac{\sum_k \mu_k^j(t) N_k^j(t)}{\sum_k N_k^j(t)}.$$

This was repeated for each sample from the posterior distribution. The resulting national trends in population mean GRB are presented in Figure S1. Further aggregation using annual national adult population totals $N^j(t) = \sum_k N_k^j(t)$ provided time-dependent posterior distributions of mean population GRB for cultural zones (and, analogously, for HDI categories, not shown here), again repeated for each sample from the posterior distribution, via

$$\mu^c(t) = \frac{\sum_{j:c[j]=c} \mu^j(t) N^j(t)}{\sum_{j:c[j]=c} N^j(t)},$$

presented in Figure 1B in the main text; and, finally, for the global population via

$$\mu(t) = \frac{\sum_j \mu^j(t) N^j(t)}{\sum_j N^j(t)},$$

presented in Figure 1A in the main text as solid line. The dashed line in Figure 1A in the main text is a simple average of countries, not weighted by population sizes, computed as

$$\bar{\mu}(t) = \frac{\sum_j \mu^j(t)}{\sum_j 1}.$$

The map in Figure 1D in the main text presents point estimates (posterior means) for the country-wise contrasts $\mu^j(2022) - \mu^j(1995)$.

[11] HDI categories were Low (< 0.55), Medium (≥ 0.55 and < 0.7), High (≥ 0.7 and < 0.8), and Very High (≥ 0.8). For a time-independent classification, we chose the year 2009 as the reference year, which was the center of the observation period. The HDI data were obtained from `https://hdr.undp.org/data-center/documentation-and-downloads` (accessed January 2025).

Further analyses

In RQ3, we explored the role of differential population growth for explaining the global trend. The question was approached by further processing the results from above HGAM with post-stratification analysis in three different ways. First, we fit the Bayesian linear regression model

$$\log\left(N^j(2022)\right) = \gamma_0 + \gamma_1\ \mu^j(1995) + \log\left(N^j(1995)\right) + \xi_j,$$

where $\log(N^j(1995))$ was an offset variable and $\xi_j \sim \mathcal{N}(0, \psi^2)$. Note that this is equivalent to a model with log factor change in adult population size as outcome variable:

$$\log\left(\frac{N^j(2022)}{N^j(1995)}\right) = \gamma_0 + \gamma_1\ \mu^j(1995) + \xi_j.$$

Since $\mu^j(1995)$ was not known, but estimated, we included it not as a fixed predictor, but as a predictor with normally distributed measurement error. Mean and SD of the measurement error for each country j were computed by taking the sample mean and sample SD from the posterior samples of $\mu^j(1995)$. The model was implemented in `brms` v2.23.0 (Bürkner, 2017) using the `mi()` function for predictors with missing values or measurement noise and the default non- (for γ_1) and weakly informative priors (for all other parameters). From the joint posterior samples of model parameters, it was possible to compute the posterior distribution of expected factor change in adult population size between 1995 and 2022 as a function of an arbitrary level x of population mean GRB in 1995 via

$$\exp(\gamma_0 + \gamma_1 x),$$

which is visualized in Figure 2A in the main text for $x \in [0, 100]$.

Second, we compared the posterior distributions of $\mu(1995)$ and $\mu(2022)$ to the posterior distribution of a counterfactual global population mean GRB in the year 2022 if national adult population sizes had remained unchanged since 1995 (i.e., the share that countries make up of the global adult population), but all else (i.e., composition of national population in terms of age and sex and the mean GRB in all demographic groups) had changed as observed. The calculation combined each country's GRB from 2022 with the population size from 1995:

$$\frac{\sum_j \mu^j(2022) N^j(1995)}{\sum_j N^j(1995)}.$$

The three distributions are presented in Figure 2B in the main text.

Third, we computed the Spearman rank correlation coefficient on the country level between population mean GRB $\mu^j(t)$ (repeatedly for each sample from the posterior distribution) and adult population size $N^j(t)$, once for the year $t = 1995$ and once for the year $t = 2022$. Posterior summaries of the correlation coefficients are presented in Figure 2C in the main text.

S5.3 The LMM

In RQ4, we asked about the association of GRB and completed fertility. This was a cross-sectional analysis of the most recent survey per country, but not older than year 2010, yielding a sample of 101 countries (identical to the sample of countries for the analysis presented in Supplementary Materials S3).

We used the WEVS item "How many children do you have?". In the recent survey waves included in this analysis, the upper limit of the response scale was "5 or more children", which we treated as "5". We restricted the sample to women of age 40 to 49. In order to be able to use WEVS data for fertility analysis, this self-reported number of children a woman *currently had* at the time of the survey was taken as proxy variable for the total number of children that were *ever born alive to her*. The average number of children born to women aged 40 to 49 years approximately estimates the completed cohort fertility rate, albeit with some potential disturbances due to mortality or migration by women before that age (Newell, 1990). Here, we used women's self-reported number of children at age 40 to 49 as a proxy for their completed fertility, that is, the number of births they have had toward the end of their reproductive window.

In the LMM, we estimated the average effect of one SD increase on average number of children both *within* and *between* countries. Both demographic processes can affect the global population GRB. The strength of these associations could differ, for example because countries with higher levels of mean GRB also differ in other, unobserved national-level characteristics associated with fertility (an instance of cluster-level confounding). This was done by group-mean centering the standardized predictor GRB (mean zero and SD one) within countries and entering both the individual-level deviation from the country mean and the country mean itself as a predictor. The mixed model then reads

$$
\begin{aligned}
&y_{ij} = \beta_0 + \mu_i + (\beta_1 + \omega_i)(\mathrm{GRB}^{\mathrm{std}}_{ij} - \overline{\mathrm{GRB}^{\mathrm{std}}}_i) + \beta_2 \overline{\mathrm{GRB}^{\mathrm{std}}}_i + \epsilon_{ij},\\
&\begin{pmatrix}\mu_i\\ \omega_i\end{pmatrix} \sim \mathcal{N}\left(\begin{pmatrix}0\\0\end{pmatrix}, \begin{pmatrix}\sigma^2_\mu & \sigma_{\mu\omega}\\ \sigma_{\mu\omega} & \sigma^2_\omega\end{pmatrix}\right),\\
&\epsilon_{ij} \sim \mathcal{N}(0, \sigma^2_\epsilon),
\end{aligned}
$$

where y_{ij} is the number of children reported by the jth woman in country i, μ_i and ω_i are the random country-specific intercept and slope, respectively, modeled as draws from a bivariate normal distribution, and $\overline{\mathrm{GRB}^{\mathrm{std}}}_i$ and $\mathrm{GRB}^{\mathrm{std}}_{ij}$ are the country mean and individual standardized GRB score, respectively. With this model, we decompose variation in the number of children of women aged 40 to 49 years into

- an average within-country effect of a woman's GRB across all countries (β_1),
- unexplained between-country variation in the within-country effect of a woman's GRB (σ^2_ω),
- the effect of the country-level average GRB (β_2),
- unexplained between-country variation (σ^2_μ), and
- unexplained within-country variation (σ^2_ϵ).

This is an example of what has been called a *hybrid* model (Townsend et al., 2013), a model specification which combines multiple advantages of both fixed-effects and random-effects models (besides allowing a separation of within and between effects). For example, country-constant predictors and country random intercepts can be included simultaneously, while we also adjust for potential violations of the random effects assumption (independence between individual-level GRB and group-level errors).

In additional models, besides age (which only varies between 40 and 49 years due to the sample restriction), we added further predictors related to demographic characteristics – education, income level (self-reported category relative to other households in the country, counting all household wages, salaries, pensions and other incomes), town size – and religiosity, of which we hypothesized they could correlate with fertility and GRB and hence would explain some of the covariation. Religiosity, for example, has been linked to views about women's role in society and fertility decision-making on a global level (Norris & Inglehart, 2004). A

person's religiosity was taken to be the arithmetic mean of four questionnaire items after min-max normalization (see also Akaliyski, 2023):

- Here is a list of qualities that children can be encouraged to learn at home. Which, if any, do you consider to be especially important? Please choose up to five!

  Religious faith chosen (100) / Religious faith not chosen (0)

- Independently of whether you attend religious services or not, would you say you are...?

  A religious person (100) / Not a religious person (50) / A convinced atheist (0)

- Apart from weddings and funerals, about how often do you attend religious services these days?

  More than once a week (100) / Once a week (83.33) / Once a month (66.67) / Only on holy days (50) / Once a year (33.33) / Less often (16.67) / Never, practically never (0)

- For each of the following, indicate how important it is in your life. Would you say it is very important, rather important, not very important, or not at all important?

  Religion very important (100) / Religion rather important (66.67) / Religion not very important (33.33) / Religion not at all important (0)

Religiosity was also standardized (mean zero and SD one) before being entered in the model. The model was fit with REML using the `lmer()` function from the `lme4` package v1.1-38 (Bates et al., 2015).

S5.4 The Bayesian hierarchical period ASFR model

RQ4 was also analyzed more extensively with the ASFR model, which allowed us to obtain estimates of total fertility rate, but also fertility timing for women with different GRB in the same country. This was a cross-sectional analysis of the most recent survey per country, but not older than year 2010, yielding a sample of 101 countries (see the sample of countries in Supplementary Materials S3 or Table S2). We included 49,433 women with age 18 to 49 years, i.e., using period data rather than cohort data.

The model built on Kostaki & Paraskevi (2007, their model 1). An ASFR model explicates the relationship between a woman's age and her expected number of children born alive at that age. Various mathematical models for this relationship have been proposed and evaluated in fertility datasets (see, e.g., Kostaki & Paraskevi, 2007; Mishra & Upadhyay, 2019). We chose a relatively simple four-parameter model based on a (non-normalized) split normal distribution: A Gaussian bell shape with one location parameter for the peak (mode), one variance parameter before and another variance parameter after the peak, and one parameter controlling the "height" of the curve (Kostaki & Paraskevi, 2007). The model struck a balance between complexity (number of parameters) and flexibility, while also providing interpretable parameters. The latter also facilitated the choice of weakly informative prior distributions (see below). The chosen model has been described as "flexible in the sense that it provides explanation to a vivid range of age-specific fertility patterns" (Mishra & Upadhyay, 2019, p. 155). This was important when applied across a diverse set of countries.

The basic ASFR model from the literature was embedded in a Bayesian hierarchical model that induced partial pooling between countries. We fit essentially two fertility curves per country, for two different subpopulations of women: Those with *less egalitarian* GRB (equal to or lower than the global sample median of 66.67) and *more egalitarian* GRB (higher than the global sample median of 66.67). Although the inclusion of the GRB variable on a continuous scale would have been preferable, the dichotomization via a median split limited the complexity of the non-linear model, which was helpful to reach convergence. Table S2 describes the resulting sample sizes per country-subpopulation cell.

As expected, given substantial between-country variation in GRB (see, e.g., Supplementary Materials S1), the relative frequency of women with *less egalitarian* GRB (equal to or lower than the global sample median of 66.67) and *more egalitarian* GRB (higher than the global sample median of 66.67) in the sample varied widely across countries. Importantly, all countries contributed samples to both subpopulations: No cells in Table S2 are unoccupied. More than half of the countries exhibited reasonable variation, with each subpopulation comprising at least 25% of the sample. Furthermore, our Bayesian hierarchical approach to parameter estimation partially mitigates the limited information available from small sample sizes in some country-subpopulation cells. Weakly informative prior distributions imposed restrictions on the model parameters in the average country, considering plausible ranges before fitting the model (see below). Further, the hierarchical nature of the model induced partial pooling of information not only for each model parameter separately (by assuming a common distribution for each b_j across countries), but also across subpopulations (via the correlation structure in $\mathbf{\Omega}$), allowing further borrowing of strength.[12] Therefore, even for country-subpopulation combinations with few observed data points, likely values of the model parameters could be inferred. Remaining uncertainty was adequately reflected in the variance of our posterior distributions of interest and depicted as part of the ASFR modeling results (e.g., Figure 3 in the main text).

[12] This is exemplified by the posterior summary of the model parameters' correlation matrix in Table S4. The high positive correlation between b_2 and b_6 across countries, for example, implies that it was possible to learn about age of highest fertility in one subpopulation from age of highest fertility in the same country's other subpopulation. This was a reasonable modeling choice, given that fertility patterns are influenced by many factors acting on the country level (e.g., Skirbekk, 2022).

Table S2: Sample sizes (number of women aged 18 to 49 years) per country and GRB subpopulation resulting from the median split. The sample is used for fitting the Bayesian hierarchical ASFR model.

Country (year)	GRB subpopulation	Sample size	Country (year)	GRB subpopulation	Sample size	Country (year)	GRB subpopulation	Sample size
Albania (2018)	Higher (> 66.67)	364 (69%)	Hong Kong SAR China (2018)	Higher (> 66.67)	376 (61%)	Peru (2018)	Higher (> 66.67)	305 (63%)
	Lower (≤ 66.67)	163 (31%)		Lower (≤ 66.67)	237 (39%)		Lower (≤ 66.67)	177 (37%)
Algeria (2014)	Higher (> 66.67)	130 (29%)	Hungary (2018)	Higher (> 66.67)	247 (66%)	Philippines (2019)	Higher (> 66.67)	64 (17%)
	Lower (≤ 66.67)	314 (71%)		Lower (≤ 66.67)	129 (34%)		Lower (≤ 66.67)	311 (83%)
Andorra (2018)	Higher (> 66.67)	253 (89%)	Iceland (2017)	Higher (> 66.67)	422 (95%)	Poland (2017)	Higher (> 66.67)	230 (75%)
	Lower (≤ 66.67)	30 (11%)		Lower (≤ 66.67)	20 (5%)		Lower (≤ 66.67)	75 (25%)
Argentina (2017)	Higher (> 66.67)	237 (77%)	India (2022)	Higher (> 66.67)	75 (24%)	Portugal (2020)	Higher (> 66.67)	183 (78%)
	Lower (≤ 66.67)	71 (23%)		Lower (≤ 66.67)	238 (76%)		Lower (≤ 66.67)	53 (22%)
Armenia (2021)	Higher (> 66.67)	142 (35%)	Indonesia (2018)	Higher (> 66.67)	136 (10%)	Puerto Rico (2018)	Higher (> 66.67)	254 (85%)
	Lower (≤ 66.67)	264 (65%)		Lower (≤ 66.67)	1239 (90%)		Lower (≤ 66.67)	44 (15%)
Australia (2018)	Higher (> 66.67)	430 (94%)	Iran (2020)	Higher (> 66.67)	114 (19%)	Qatar (2010)	Higher (> 66.67)	42 (9%)
	Lower (≤ 66.67)	26 (6%)		Lower (≤ 66.67)	478 (81%)		Lower (≤ 66.67)	429 (91%)
Austria (2018)	Higher (> 66.67)	346 (85%)	Iraq (2018)	Higher (> 66.67)	61 (13%)	Romania (2018)	Higher (> 66.67)	365 (50%)
	Lower (≤ 66.67)	61 (15%)		Lower (≤ 66.67)	415 (87%)		Lower (≤ 66.67)	371 (50%)
Azerbaijan (2018)	Higher (> 66.67)	130 (23%)	Italy (2018)	Higher (> 66.67)	394 (76%)	Russia (2017)	Higher (> 66.67)	367 (34%)
	Lower (≤ 66.67)	427 (77%)		Lower (≤ 66.67)	123 (24%)		Lower (≤ 66.67)	712 (66%)
Bangladesh (2018)	Higher (> 66.67)	69 (14%)	Japan (2019)	Higher (> 66.67)	95 (46%)	Rwanda (2012)	Higher (> 66.67)	180 (26%)
	Lower (≤ 66.67)	428 (86%)		Lower (≤ 66.67)	110 (54%)		Lower (≤ 66.67)	516 (74%)
Belarus (2018)	Higher (> 66.67)	219 (49%)	Jordan (2018)	Higher (> 66.67)	43 (11%)	Serbia (2018)	Higher (> 66.67)	310 (70%)
	Lower (≤ 66.67)	232 (51%)		Lower (≤ 66.67)	346 (89%)		Lower (≤ 66.67)	130 (30%)
Bolivia (2017)	Higher (> 66.67)	416 (55%)	Kazakhstan (2018)	Higher (> 66.67)	110 (24%)	Singapore (2020)	Higher (> 66.67)	372 (65%)
	Lower (≤ 66.67)	346 (45%)		Lower (≤ 66.67)	341 (76%)		Lower (≤ 66.67)	196 (35%)
Bosnia & Herzegovina (2019)	Higher (> 66.67)	279 (55%)	Kenya (2021)	Higher (> 66.67)	281 (49%)	Slovakia (2022)	Higher (> 66.67)	102 (43%)
	Lower (≤ 66.67)	224 (45%)		Lower (≤ 66.67)	292 (51%)		Lower (≤ 66.67)	137 (57%)
Brazil (2018)	Higher (> 66.67)	410 (75%)	Kuwait (2014)	Higher (> 66.67)	78 (21%)	Slovenia (2017)	Higher (> 66.67)	217 (82%)
	Lower (≤ 66.67)	138 (25%)		Lower (≤ 66.67)	291 (79%)		Lower (≤ 66.67)	49 (18%)
Bulgaria (2017)	Higher (> 66.67)	193 (57%)	Kyrgyzstan (2020)	Higher (> 66.67)	68 (14%)	South Africa (2013)	Higher (> 66.67)	494 (37%)
	Lower (≤ 66.67)	144 (43%)		Lower (≤ 66.67)	402 (86%)		Lower (≤ 66.67)	839 (63%)
Canada (2020)	Higher (> 66.67)	1081 (89%)	Latvia (2021)	Higher (> 66.67)	214 (65%)	South Korea (2018)	Higher (> 66.67)	112 (32%)
	Lower (≤ 66.67)	127 (11%)		Lower (≤ 66.67)	114 (35%)		Lower (≤ 66.67)	238 (68%)
Chile (2018)	Higher (> 66.67)	170 (54%)	Lebanon (2018)	Higher (> 66.67)	151 (35%)	Spain (2017)	Higher (> 66.67)	285 (90%)
	Lower (≤ 66.67)	142 (46%)		Lower (≤ 66.67)	282 (65%)		Lower (≤ 66.67)	33 (10%)
China (2018)	Higher (> 66.67)	456 (45%)	Libya (2022)	Higher (> 66.67)	73 (19%)	Sweden (2017)	Higher (> 66.67)	256 (95%)
	Lower (≤ 66.67)	561 (55%)		Lower (≤ 66.67)	311 (81%)		Lower (≤ 66.67)	13 (5%)
Colombia (2018)	Higher (> 66.67)	399 (70%)	Lithuania (2018)	Higher (> 66.67)	217 (59%)	Switzerland (2017)	Higher (> 66.67)	711 (84%)
	Lower (≤ 66.67)	168 (30%)		Lower (≤ 66.67)	150 (41%)		Lower (≤ 66.67)	134 (16%)
Croatia (2017)	Higher (> 66.67)	325 (76%)	Macao SAR China (2019)	Higher (> 66.67)	144 (53%)	Taiwan (2019)	Higher (> 66.67)	198 (62%)
	Lower (≤ 66.67)	104 (24%)		Lower (≤ 66.67)	128 (47%)		Lower (≤ 66.67)	122 (38%)
Cyprus (2019)	Higher (> 66.67)	137 (46%)	Malaysia (2018)	Higher (> 66.67)	150 (31%)	Tajikistan (2020)	Higher (> 66.67)	83 (21%)
	Lower (≤ 66.67)	161 (54%)		Lower (≤ 66.67)	338 (69%)		Lower (≤ 66.67)	314 (79%)
Czechia (2022)	Higher (> 66.67)	171 (51%)	Maldives (2021)	Higher (> 66.67)	192 (44%)	Thailand (2018)	Higher (> 66.67)	131 (38%)
	Lower (≤ 66.67)	164 (49%)		Lower (≤ 66.67)	240 (56%)		Lower (≤ 66.67)	217 (62%)
Denmark (2017)	Higher (> 66.67)	678 (94%)	Mexico (2018)	Higher (> 66.67)	304 (54%)	Trinidad & Tobago (2010)	Higher (> 66.67)	196 (70%)
	Lower (≤ 66.67)	43 (6%)		Lower (≤ 66.67)	256 (46%)		Lower (≤ 66.67)	84 (30%)
Ecuador (2018)	Higher (> 66.67)	261 (57%)	Mongolia (2020)	Higher (> 66.67)	136 (22%)	Tunisia (2019)	Higher (> 66.67)	89 (24%)
	Lower (≤ 66.67)	194 (43%)		Lower (≤ 66.67)	492 (78%)		Lower (≤ 66.67)	282 (76%)
Egypt (2018)	Higher (> 66.67)	37 (9%)	Montenegro (2019)	Higher (> 66.67)	144 (50%)	Turkey (2018)	Higher (> 66.67)	251 (30%)
	Lower (≤ 66.67)	389 (91%)		Lower (≤ 66.67)	145 (50%)		Lower (≤ 66.67)	576 (70%)
Estonia (2018)	Higher (> 66.67)	174 (74%)	Morocco (2021)	Higher (> 66.67)	201 (40%)	Ukraine (2020)	Higher (> 66.67)	447 (50%)
	Lower (≤ 66.67)	61 (26%)		Lower (≤ 66.67)	296 (60%)		Lower (≤ 66.67)	445 (50%)
Ethiopia (2020)	Higher (> 66.67)	217 (39%)	Myanmar (Burma) (2020)	Higher (> 66.67)	53 (11%)	United Kingdom (2018)	Higher (> 66.67)	390 (88%)
	Lower (≤ 66.67)	337 (61%)		Lower (≤ 66.67)	411 (89%)		Lower (≤ 66.67)	54 (12%)
Finland (2017)	Higher (> 66.67)	209 (96%)	Netherlands (2022)	Higher (> 66.67)	377 (92%)	United States (2017)	Higher (> 66.67)	736 (82%)
	Lower (≤ 66.67)	9 (4%)		Lower (≤ 66.67)	32 (8%)		Lower (≤ 66.67)	164 (18%)
France (2018)	Higher (> 66.67)	388 (88%)	New Zealand (2020)	Higher (> 66.67)	158 (95%)	Uruguay (2022)	Higher (> 66.67)	268 (89%)
	Lower (≤ 66.67)	52 (12%)		Lower (≤ 66.67)	9 (5%)		Lower (≤ 66.67)	34 (11%)
Georgia (2018)	Higher (> 66.67)	238 (40%)	Nicaragua (2020)	Higher (> 66.67)	251 (54%)	Uzbekistan (2022)	Higher (> 66.67)	53 (12%)
	Lower (≤ 66.67)	360 (60%)		Lower (≤ 66.67)	211 (46%)		Lower (≤ 66.67)	407 (88%)
Germany (2018)	Higher (> 66.67)	312 (90%)	Nigeria (2018)	Higher (> 66.67)	111 (20%)	Venezuela (2021)	Higher (> 66.67)	307 (64%)
	Lower (≤ 66.67)	35 (10%)		Lower (≤ 66.67)	437 (80%)		Lower (≤ 66.67)	169 (36%)
Ghana (2012)	Higher (> 66.67)	175 (25%)	North Macedonia (2019)	Higher (> 66.67)	204 (67%)	Vietnam (2020)	Higher (> 66.67)	160 (30%)
	Lower (≤ 66.67)	514 (75%)		Lower (≤ 66.67)	101 (33%)		Lower (≤ 66.67)	367 (70%)
Greece (2017)	Higher (> 66.67)	187 (63%)	Norway (2018)	Higher (> 66.67)	302 (95%)	Yemen (2014)	Higher (> 66.67)	46 (12%)
	Lower (≤ 66.67)	110 (37%)		Lower (≤ 66.67)	16 (5%)		Lower (≤ 66.67)	348 (88%)
Guatemala (2020)	Higher (> 66.67)	322 (65%)	Pakistan (2018)	Higher (> 66.67)	65 (8%)	Zimbabwe (2020)	Higher (> 66.67)	196 (44%)
	Lower (≤ 66.67)	174 (35%)		Lower (≤ 66.67)	764 (92%)		Lower (≤ 66.67)	252 (56%)
Haiti (2016)	Higher (> 66.67)	325 (38%)	Palestinian Territories (2013)	Higher (> 66.67)	78 (18%)			
	Lower (≤ 66.67)	522 (62%)		Lower (≤ 66.67)	347 (82%)			

We denote the ASFR at age x of a woman in country i and GRB subpopulation $k \in \{0, 1\}$ (0: less egalitarian GRB; 1: more egalitarian GRB) by $f_i^k(x)$. It is modeled non-linearly following Kostaki & Paraskevi (2007, their model 1) as

$$f_i^k(x) = c_i^k \exp\left[-\left(\frac{x - \mu_i^k}{\sigma_i^k(x)}\right)^2\right],$$
$$\sigma_i^k(x) = \sigma_{1i}^k \text{ if } x \leq \mu_i^k, \text{ and } \sigma_i^k(x) = \sigma_{2i}^k \text{ if } x > \mu_i^k, \tag{1}$$

where c_i^k (a scaling factor that controls the "height" of the curve, i.e., closely linked to the total fertility rate), μ_i^k (location of the peak of the curve, i.e., age at which fertility is highest), σ_{1i}^k (gentleness of the fertility increase before the peak of the curve), and σ_{2i}^k (gentleness of the fertility decrease after the peak of the curve) are the four parameters of the fertility curve described by the function $f_i^k(x)$. The hierarchical nature of the model was specified via

$$\boldsymbol{b}_i = (b_{1i}, b_{2i}, b_{3i}, b_{4i}, b_{5i}, b_{6i}, b_{7i}, b_{8i})^\top \sim \mathcal{N}_8(\boldsymbol{\beta}, \boldsymbol{\Sigma}),$$

with

$$\begin{aligned}
c_i^0 &= \exp(b_{1i}),\\
\mu_i^0 &= \exp(b_{2i}),\\
\sigma_{1i}^0 &= \exp(b_{3i}),\\
\sigma_{2i}^0 &= \exp(b_{4i}),\\
c_i^1 &= \exp(b_{5i}),\\
\mu_i^1 &= \exp(b_{6i}),\\
\sigma_{1i}^1 &= \exp(b_{7i}),\\
\sigma_{2i}^1 &= \exp(b_{8i}).
\end{aligned}$$

That is, the country-specific parameters $\boldsymbol{b}_i$, defining the fertility curve in country i, were assumed to be random effects from a multivariate Normal distribution. The log transformation ensured positive parameter values. The curve parameters for the average country are obtained as $c^0 = \exp(\beta_1)$, $\mu^0 = \exp(\beta_2)$, $\sigma_1^0 = \exp(\beta_3)$, and $\sigma_2^0 = \exp(\beta_4)$ for $k = 0$ (i.e., the subpopulation with less egalitarian GRB), and analogously using β_5 through β_8 for $k = 1$.

We did not evaluate other models from the literature. We expected that different models will fit some "types" of fertility patterns in our global sample better, but others worse – likely without much consequences for the main conclusions. Patterns our model cannot handle, for example, are bimodal fertility curves, or curves with multiple "humps" at earlier and later ages due to, for example, different subgroups in the population or ongoing demographic transitions (Burkimsher, 2017; Kostaki & Paraskevi, 2007). But note that the subpopulation split along women's GRB is likely to capture a relevant share of within-country differences in fertility timing – thereby reducing the flexibility required for the basic ASFR model to account for excess heterogeneity.

As we relied on the WEVS, we could not use the standard type of datasets conventionally used for fitting ASFR models. Typically, these are tables with age brackets and number of live births per 1,000 women in that age bracket for a given period and population. But the WEVS data contained no information about age at childbirth. We could only observe how many children have been received by a woman so far – again, like for the LMM, using the total number of children a woman *currently had* at the time of the survey as proxy variable for the total number of children who were *ever born alive to her*. However, the key insight behind our approach was that the number of children a woman had at her current age was governed by the sum

of all age-specific fertilities for the ages she had already experienced during her life course. Information on these "earlier" age-specific fertilities, in turn, was provided by younger women from the same period (i.e., in our case, in the same survey sample). This principle enabled us to investigate the relationship between GRB and period fertility curves, using only WEVS data. Hence, we linked the above model in equation 1 to the data on reported number of children by extending the likelihood in the following way:

$$\begin{aligned} \lambda_i^k(x) &= \int_0^x f_i^k(a)\, da, \\ y_{i,x}^k &\sim \text{Poisson}\left(\lambda_i^k(x) \cdot n_{i,x}^k\right) \end{aligned} \tag{2}$$

where $y_{i,x}^k$ is the sum of all children in country i's subpopulation k of age x, and $n_{i,x}^k$ is the respective number of women. With this approach via the cumulative ASFR $\lambda_i^k(x)$, it was possible to estimate the parameters in equation 1 using only data on $y_{i,x}^k$ and $n_{i,x}^k$ from WEVS.

The Bayesian analysis enabled us to put prior distributions on parameters $\boldsymbol{\beta}$ and $\boldsymbol{\Sigma}$. We chose weakly informative priors in order to induce some weak regularization and stabilization of estimation. This was different from previous Bayesian work using non-informative priors for parameters of ASFR models (Mishra & Upadhyay, 2019). We deemed this appropriate given the large number of parameters whose joint posterior distribution needed to be inferred with rather limited data. The priors were intended to reduce the probability of parameter values in clearly unrealistic, or even biologically impossible ranges (e.g., a fertility peak at very high or very old biological ages). Such control was possible, since $\boldsymbol{\beta}$ contained well-interpretable parameters (see equation 1). To ground the plausible range implied by the priors in further scientific knowledge, we consulted the previous literature that either used the same basic four-parameter ASFR model as we did or a closely related model with one additional parameter, and inspected their parameter estimates. This included models fitted to historical data from multiple European countries (Kostaki & Paraskevi, 2007; Mishra & Upadhyay, 2019), multiple African countries (Gayawan et al., 2010) and India (Srivastava et al., 2021). We used the same prior distributions for ASFR parameters of both GRB subpopulations to not tune our model in a way favoring an effect of GRB.

The priors were

$$\begin{aligned} \boldsymbol{\beta} &\sim \mathcal{N}_8\left(\begin{pmatrix} \log(0.125) \\ \log(25) \\ \log(5) \\ \log(5) \\ \log(0.125) \\ \log(25) \\ \log(5) \\ \log(5) \end{pmatrix}, \text{diag}\begin{pmatrix} 0.5^2 \\ 0.2^2 \\ 0.75^2 \\ 0.75^2 \\ 0.5^2 \\ 0.2^2 \\ 0.75^2 \\ 0.75^2 \end{pmatrix}\right), \\ \boldsymbol{\Sigma} &= \text{diag}(\boldsymbol{\tau}) \cdot \boldsymbol{\Omega} \cdot \text{diag}(\boldsymbol{\tau}), \\ \boldsymbol{\Omega} &\sim \text{LKJ}(1), \\ \boldsymbol{\tau} &\sim \text{Half-}\mathcal{N}_8\left(\mathbf{0}, \text{diag}\begin{pmatrix} 0.5^2 \\ 0.2^2 \\ 0.75^2 \\ 0.75^2 \\ 0.5^2 \\ 0.2^2 \\ 0.75^2 \\ 0.75^2 \end{pmatrix}\right). \end{aligned}$$

For example, this implied a prior range (95% prior probability) for the age of highest fertility in any subpopulation in the average country of

$$\exp\left(\log(25) \pm 1.96 \cdot 0.2\right) = \{16.9; 37.0\}.$$

This range contains estimated ages of peak fertility in countries as diverse as India and Denmark. Ages lower than 17 years or ages higher than 37 years constitute implausible population fertility peaks. Similarly, the scaling parameter c (see equation 1) typically takes values roughly between 0.1 (lower fertility countries) and 0.25 (higher fertility countries) in the literature. Our priors imply a range in any subpopulation in the average country of

$$\exp\left(\log(0.125) \pm 1.96 \cdot 0.5\right) = \{0.05; 0.33\},$$

thus providing a reasonable, yet soft restriction. Note that extreme values for some countries and their subpopulations beyond this range are not excluded. This is because individual country parameters $\boldsymbol{b}_i$ have larger prior variance than the average-country parameter $\boldsymbol{\beta}$, given our hierarchical prior for $\boldsymbol{\Sigma}$ that allows for between-country heterogeneity.

The model was fit via MCMC in Stan v2.37 (Carpenter et al., 2017) using the `cmdstanr` interface v0.9.0 (Gabry et al., 2025). The model was implemented in a non-centered reparameterization and using a summation over discrete age values, in line with the representation of ages in the data, in place of the integration shown in equation 2. We sampled 2,000 draws from the joint posterior using two chains (1,000 posterior draws per chain) after 1,000 warm-up iterations each with the NUTS algorithm using the sampler setting `adapt_delta = 0.99` and default maximum tree depth 10. Convergence and mixing were checked with the R hat diagnostic, trace plots and autocorrelation plots, which all indicated no serious sampling issues; neither did Stan's sampler diagnostics indicate any problems (0 divergences, 0 occurrences of maximum tree depth, E-BFMI values 0.782, 0.728 for the two chains).

Posterior means for the cumulative period age-specific fertility rates by country are shown in Supplementary Materials S3. By plotting them against observed values, they also allow judgments about model fit. The posterior distribution of the fertility curve for the average country (i.e., based on the posterior draws from $\boldsymbol{\beta}$, the vector of means of the random effects) is visualized in Figure 3A in the main text.

Table S3 shows posterior means for all components of $\exp(\boldsymbol{\beta})$. These are the ASFR model parameters for the average country after back-transforming to the original scale.

Table S3: Posterior means of ASFR model parameters for the average country.

c^0	μ^0	σ_1^0	σ_2^0	c^1	μ^1	σ_1^1	σ_2^1
0.14	25.09	5.40	12.24	0.14	27.71	6.66	9.99

Table S4 shows posterior means for each cell of the correlation matrix $\boldsymbol{\Omega}$.

Table S4: Posterior mean correlations of country-specific ASFR model parameters.

	b_1	b_2	b_3	b_4	b_5	b_6	b_7	b_8
b_1	1.00	-0.57	-0.66	-0.24	0.80	-0.54	-0.61	0.16
b_2	-0.57	1.00	0.48	-0.01	-0.46	0.84	0.38	-0.26
b_3	-0.66	0.48	1.00	0.26	-0.40	0.34	0.70	0.09
b_4	-0.24	-0.01	0.26	1.00	-0.02	-0.01	0.14	0.71
b_5	0.80	-0.46	-0.40	-0.02	1.00	-0.56	-0.69	0.12
b_6	-0.54	0.84	0.34	-0.01	-0.56	1.00	0.59	-0.36
b_7	-0.61	0.38	0.70	0.14	-0.69	0.59	1.00	-0.05
b_8	0.16	-0.26	0.09	0.71	0.12	-0.36	-0.05	1.00

Other interesting quantities could be derived from the model's posterior by running the respective calculations repeatedly on the MCMC samples, like

- the posterior distribution of total fertility rate for a subpopulation k (either for the average country, or country i), which was simply the area under the posterior fertility curve for the childbearing ages, i.e.,

$$\int_0^{49} f_i^k(x)\, dx,$$

- the posterior distribution of age at which average fertility equaled 0.5 (one child per two women[13]) for a subpopulation k (either for the average country, or country i), which was simply the age x^* at which the area under the curve added up to exactly 0.5, i.e.,

$$x^* \text{ such that } \int_0^{x^*} f_i^k(a)\, da = 0.5,$$

- the posterior distribution of the proportion of countries for which it held that the low-GRB subpopulation had higher fertility, and
- the posterior distribution of the proportion of countries for which it held that the low-GRB subpopulation had a younger age at which average fertility equaled 0.5.

[13]The average fertility of 0.5 was chosen as a reference for comparison of fertility timing to ensure that all countries could be compared to the same standard, including very low-fertility countries, where posterior distributions put a considerable probability on values of total fertility rate < 1.

S6 Sensitivity analysis results

We assessed the robustness of our results from the longitudinal analysis (Figure 1 in the main text) to variation in sample inclusion criteria. In this sensitivity analysis, a country was only part of the longitudinal analysis if it had ≥ 3 national surveys that jointly covered at least half of the observation period (i.e., 14 years). This is stricter than the criteria applied in the main analysis (≥ 2 national surveys that jointly covered at least a quarter of the observation period, see Supplementary Materials S5.2). Thus, the sensitivity analysis only relied on countries which had been sampled more extensively over time. Otherwise, the estimation approach via the HGAM with post-stratification was the same as described in Supplementary Materials S5.2.

The restricted sample consisted of data from 321,863 respondents from 60 countries, which made up 79.7% of the world's adult (18+ years) population in 2022. Specifically, the following countries included in the main analysis (see Table S1) were no longer part of the sensitivity analysis: Algeria, Andorra, Croatia, Cyprus, Ethiopia, France, Guatemala, Hong Kong SAR China, Italy, Kazakhstan, Latvia, Libya, Lithuania, Malaysia, Moldova, Switzerland, Thailand, Uzbekistan.

Figure S24 mirrors panels A, B, C from Figure 1 in the main text, but with sensitivity analysis results. Reassuringly, conclusions about global, average-country, and group-specific time trends in mean GRB remain largely unchanged with the restricted sample.

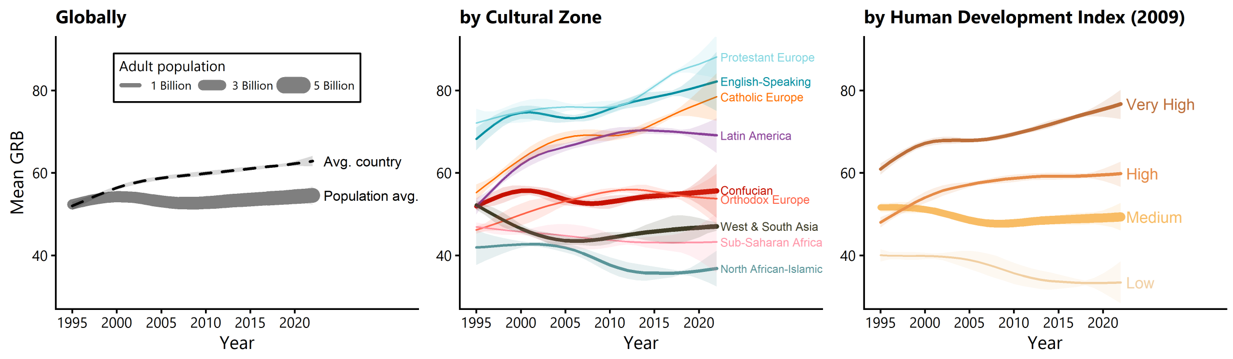

Figure S24: Results of the sensitivity analysis on trends in average GRB. See the caption of Figure 1 in the main text for further descriptions of panels A, B, C.

References

Akaliyski, P. (2023). Distinct Conceptions of Freedom in East Asia and the Protestant West Underpin Unique Pathways of Societal Development. *Journal of Cross-Cultural Psychology*, *54*(2), 173–194. https://doi.org/10.1177/00220221221143320

Anderson, C. J., Bol, D., & Ananda, A. (2021). Humanity's Attitudes about Democracy and Political Leaders: Patterns and Trends. *Public Opinion Quarterly*, *85*(4), 957–986. https://doi.org/10.1093/poq/nfab056

Bates, D., Mächler, M., Bolker, B., & Walker, S. (2015). Fitting Linear Mixed-Effects Models Using Lme4. *Journal of Statistical Software*, *67*(1), 1–48. https://doi.org/10.18637/jss.v067.i01

Boer, D., Hanke, K., & He, J. (2018). On Detecting Systematic Measurement Error in Cross-Cultural Research: A Review and Critical Reflection on Equivalence and Invariance Tests. *Journal of Cross-Cultural Psychology*, *49*(5), 713–734. https://doi.org/10.1177/0022022117749042

Bratt, C. (2025). Benefits of a Pragmatic Approach: Rethinking Measurement Invariance and Composite Scores in Cross-Cultural Research. *Sociological Methods & Research*, 00491241251405869. https://doi.org/10.1177/00491241251405869

Brown, T. A. (2015). *Confirmatory factor analysis for applied research* (2nd ed.). The Guilford Press.

Burkimsher, M. (2017). Evolution of the shape of the fertility curve: Why might some countries develop a bimodal curve? *Demographic Research*, *37*, 295–324.

Bürkner, P.-C. (2017). Brms: An R Package for Bayesian Multilevel Models Using Stan. *Journal of Statistical Software*, *80*(1), 1–28. https://doi.org/10.18637/jss.v080.i01

Carpenter, B., Gelman, A., Hoffman, M. D., Lee, D., Goodrich, B., Betancourt, M., Brubaker, M., Guo, J., Li, P., & Riddell, A. (2017). Stan: A Probabilistic Programming Language. *Journal of Statistical Software*, *76*(1), 1–32. https://doi.org/10.18637/jss.v076.i01

Charles, M. (2020). Gender Attitudes in Africa: Liberal Egalitarianism Across 34 Countries. *Social Forces*, *99*(1), 86–125. https://doi.org/10.1093/sf/soz132

Claassen, C. (2019). Estimating Smooth Country–Year Panels of Public Opinion. *Political Analysis*, *27*(1), 1–20. https://doi.org/10.1017/pan.2018.32

Eriksson, K., Björnstjerna, M., & Vartanova, I. (2020). The Relation Between Gender Egalitarian Values and Gender Differences in Academic Achievement. *Frontiers in Psychology*, *11*. https://doi.org/10.3389/fpsyg.2020.00236

Fike, R. (2024). *Economic Freedom and Gender Norms*. Fraser Institute.

Foa, R. S., Mounk, Y., & Klassen, A. (2022). Why the Future Cannot Be Predicted. *Journal of Democracy*, *33*(1), 147–155.

Gabry, J., Cesnovar, R., Johnson, A., & Bronder, S. (2025). *Cmdstanr: R Interface to 'CmdStan'* [Manual].

Gayawan, E., Adebayo, S. B., Ipinyomi, R. A., & Oyejola, B. A. (2010). Modeling fertility curves in Africa. *Demographic Research*, *22*, 211–236.

Gelman, A., & Little, T. C. (1997). Poststratification into many categories using hierarchical logistic regression. *Survey Methodology*, *23*(2), 127–135.

Ghitza, Y., & Gelman, A. (2013). Deep Interactions with MRP: Election Turnout and Voting Patterns Among Small Electoral Subgroups. *American Journal of Political Science*, *57*(3), 762–776. https://doi.org/10.1111/ajps.12004

Inglehart, R., Haerpfer, C., Moreno, A., Welzel, C., Kizilova, K., Diez-Medrano, J., Lagos, M., Norris, P., Ponarin, E., & Puranen, B. (Eds.). (2024). *World Values Survey: All Rounds - Country-Pooled Datafile*. JD Systems Institute & WVSA Secretariat. Dataset Version 5.0.0. https://doi.org/10.14281/18241.25

Kołczyńska, M., & Bürkner, P.-C. (2024). Modeling Public Opinion Over Time: A Simulation Study of Latent Trend Models. *Journal of Survey Statistics and Methodology*, *12*(1), 130–154. https://doi.org/10.1093/jssam/smad024

Kołczyńska, M., Bürkner, P.-C., Kennedy, L., & Vehtari, A. (2024). Modeling Public Opinion Over Time and Space: Trust in State Institutions in Europe, 1989-2019. *Survey Research Methods*, *18*(1), 1–19. https://doi.org/10.18148/srm/2024.v18i1.8119

Kostaki, A., & Paraskevi, P. (2007). Modeling fertility in modern populations. *Demographic Research*, *16*, 141–194. https://doi.org/10.4054/DemRes.2007.16.6

Kusano, K., Napier, J. L., & Jost, J. T. (2025). The Mismeasure of Culture: Why Measurement Invariance Is Rarely Appropriate for Comparative Research in Psychology. *Personality and Social Psychology Bulletin*, 01461672251341402. https://doi.org/10.1177/01461672251341402

Lax, J. R., & Phillips, J. H. (2009). How Should We Estimate Public Opinion in The States? *American Journal of Political Science*, *53*(1), 107–121. https://doi.org/10.1111/j.1540-5907.2008.00360.x

Minkov, M., Vignoles, V. L., Welzel, C., Akaliyski, P., Bond, M. H., Kaasa, A., & Smith, P. B. (2024). Comparative Culturology

and Cross-Cultural Psychology: How Comparing Societal Cultures Differs From Comparing Individuals' Minds Across Cultures. *Journal of Cross-Cultural Psychology*, *55*(2), 164–188. https://doi.org/10.1177/00220221231220027

Mishra, R. K., & Upadhyay, S. K. (2019). Parametric Bayes Analyses to Study the Age-Specific Fertility Patterns. *American Journal of Mathematical and Management Sciences*, *38*(2), 151–173. https://doi.org/10.1080/01966324.2018.1491355

Newell, C. (1990). *Methods and models in demography*. The Guilford Press.

Norris, P., & Inglehart, R. (2004). *Sacred and secular: Religion and politics worldwide*. Cambridge University Press.

Ogden, H. (2024). *Flexible Models for Simple Longitudinal Data* (No. arXiv:2401.11827). arXiv. https://doi.org/10.48550/arXiv.2401.11827

Park, D. K., Gelman, A., & Bafumi, J. (2004). Bayesian Multilevel Estimation with Poststratification: State-Level Estimates from National Polls. *Political Analysis*, *12*(4), 375–385. https://doi.org/10.1093/pan/mph024

Pedersen, E. J., Miller, D. L., Simpson, G. L., & Ross, N. (2019). Hierarchical generalized additive models in ecology: An introduction with mgcv. *PeerJ*, *7*, e6876. https://doi.org/10.7717/peerj.6876

R Core Team. (2025). *R: A Language and Environment for Statistical Computing*. R Foundation for Statistical Computing.

Rosseel, Y. (2012). lavaan: An R package for structural equation modeling. *Journal of Statistical Software*, *48*(2), 1–36. https://doi.org/10.18637/jss.v048.i02

Simpson, G. L. (2024). gratia: An R package for exploring generalized additive models. *Journal of Open Source Software*, *9*(104), 6962. https://doi.org/10.21105/joss.06962

Skirbekk, V. (2022). *Decline and Prosper! Changing Global Birth Rates and the Advantages of Fewer Children*. Palgrave Macmillan. https://doi.org/10.1007/978-3-030-91611-4

Sokolov, B. (2018). The Index of Emancipative Values: Measurement Model Misspecifications. *American Political Science Review*, *112*(2), 395–408. https://doi.org/10.1017/S0003055417000624

Solt, F. (2020). *Modeling Dynamic Comparative Public Opinion*. https://doi.org/10.31235/osf.io/d5n9p

Srivastava, U., Singh, K. K., Pandey, A., & Narayan, N. (2021). Experiments in modeling recent Indian fertility pattern. *Scientific Reports*, *11*(1), 6592. https://doi.org/10.1038/s41598-021-85959-z

Townsend, Z., Buckley, J., Harada, M., & Scott, M. A. (2013). The Choice Between Fixed and Random Effects. In *The SAGE Handbook of Multilevel Modeling* (1st ed., pp. 73–88). SAGE Publications Ltd.

Welzel, C. (2013). *Freedom Rising: Human Empowerment and the Quest for Emancipation*. Cambridge University Press. https://doi.org/10.1017/CBO9781139540919

Welzel, C., Brunkert, L., Kruse, S., & Inglehart, R. F. (2023). Non-invariance? An Overstated Problem With Misconceived Causes. *Sociological Methods & Research*, *52*(3), 1368–1400. https://doi.org/10.1177/0049124121995521

Welzel, C., & Inglehart, R. F. (2016). Misconceptions of Measurement Equivalence: Time for a Paradigm Shift. *Comparative Political Studies*, *49*(8), 1068–1094. https://doi.org/10.1177/0010414016628275

Wood, S. N. (2017). *Generalized Additive Models: An Introduction with R* (2nd ed.). Chapman and Hall/CRC.

Zhirkov, K., & Welzel, C. (2024). Rethinking Measurement Equivalence in Comparative Political Research. *Political Research Quarterly*, *77*(3), 696–712. https://doi.org/10.1177/10659129241241753
